# Comprehensive chemical and bioactive investigation of Chinese peony flower: a case of valorization of by-products as a new food ingredient from Chinese herb

**DOI:** 10.3389/fpls.2024.1501966

**Published:** 2025-01-27

**Authors:** Meng-ling Peng, Ming-Jiong Gong, Jing Zhang, Anastassiya V. Gadetskaya, Qian-Wen Liang, Pei-Wen He, Xiao-Hui Qiu, Zhi-Hai Huang, Wen Xu

**Affiliations:** ^1^ State Key Laboratory of Traditional Chinese Medicine Syndrome, The Second Affiliated Hospital of Guangzhou University of Chinese Medicine, Guangzhou, China; ^2^ School of Chemistry and Chemical Technology, Al-Farabi Kazakh National University, Almaty, Kazakhstan; ^3^ Chinese Medicine Guangdong Laboratory, Hengqin, China

**Keywords:** *Paeonia lactiflora*, Orbitrap, flavonoids, antioxidants, biowaste valorization

## Abstract

**Introduction:**

In the present study, the flower of Chinese peony (CPF), major waste by-product of Chinese Herb *Radix paeoniae*, was comprehensively investigated for the first time.

**Methods:**

A validated UHPLC Orbitrap Mass spectrometry combined a three-levels characterization strategy were used to analyze CPF samples from four representative cultivars. The anti-inflammatory and antioxidant activities were analyzed using RAW264.7 cells, and DPPH, ABTS, FRAP, and ORAC antioxidant assays.

**Results:**

A total of 150 chemical components were identified in CPF, among them, more than 50 components were reported from this species for the first time, with potential new chemicals reported. 67 quantified or semi-quantified targeted metabolomics analysis indicated a clear distinction between flower parts and four cultivars. CPF demonstrated significant antioxidant activities and displayed anti-inflammatory effects by reducing nitric oxide, IL-6, and TNF-a release in LPS-induced macrophages. Correlation analysis highlighted a strong positive correlation between total phenolic content and DPPH ABTS, and FRAP antioxidant activities.

**Discussion:**

The present study is the first to comprehensively investigate the chemical profile and bioactivities of CPF, which provide insights into further understanding of its health-promoting potential.

## 1 Introduction

Chinese peony (*Paeonia lactiflora* Pall.), is the most common herbaceous peony species in China, and is renowned worldwide as a time-honored ornamental and medicinal herb (Yang et al., 2023). The root of Chinese peony, known as *Radix Paeoniae*, has been commonly used as a traditional medicine in East Asia for over 1200 years for treatment of rheumatoid arthritis, systemic lupus erythematosus, hepatitis, dysmenorrhea, muscle spasms, and so on (Han et al., 2022; Xu et al., 2022; Sang et al., 2023; Wu et al., 2023).

Due to the high clinical demand for *Radix Paeoniae* in Traditional Chinese Medicine (TCM), Chinese peony is usually grown for 3-5 years to obtain the roots. However, the aerial parts are often discarded. Especially, the flowers and buds must be removed during the annual flowering period to promote root growth, causing huge waste that could be potentially valorized.

The Chinese peony flowers (CPF), also known as the ‘minister of flowers’, is one of the popular ornamental flowers, offering a rich array of colors. Beyond that, CPF is also appreciated for its potential health-promoting effects (Kang et al., 2020). In western countries, the petals of Chinese peony are parboiled with a pinch of sugar and then used in desserts or home baking, or as a dressing for fruit salads. Additionally, CPF can be utilized in the creation of cocktails and lemonades (Liu et al., 2023). In China, CPF is used as a flavorful ingredient in traditional Chinese cuisine (Yuan, 2011). However, these applications are primarily limited to small-scale usage. Recently, scented tea and its related beverage products have been attracted enormous attentions and have become very popular around the world due to not only its natural and flavorful properties, but also receiving potential health benefits (Yuan, 2011; Zhao et al., 2023). Scented tea (An et al., 2022) is a kind of tea made by brewing flowers, leaves, or herbs, and is a kind of reprocessed tea endemic to China (Yun-Jie et al., 2019). Being rich in flavonoids and anthocyanins, scented tea is claimed to have anti-aging, cardiovascular protection, and metabolism promotion effects. CPF tea is currently available on the market sporadically. Its petals, stamens, whole flowers and sprouts, individually or commonly can be made into teas with multiple production processes.

For a long time, the chemical and pharmacological research on Chinese peony has mostly been restricted to the roots, yet rarely addressed to the aerial parts. A few studies have been made focusing on the analysis of certain components of CPF, which showed that it contains large portions of polyphenols (Shu et al., 2014; Ogawa et al., 2015) and flavonoids with potential antioxidant activity. In a recent study from Liu et al (Liu et al., 2022)., a total 1102 metabolites were preliminarily annotated using Kyoto Encyclopedia of Genes and Genomes (KEGG) and compared in different parts of *P. lactiflora* flower. Li et al (Li et al., 2023) compared peony petals from 12 different varieties using LC-MS-based fingerprints and metabolomics technology. Regarding its bio-activities, studies also demonstrated CPF has anti-melanin production activity (Liu et al., 2022), and inhibits H_2_O_2_-induced cell damage (Liu et al., 2023) by upregulating the expression of nuclear Factor E2-related factor (Nrf2).

Liquid chromatography coupled with high-resolution mass spectrometer (LC-HR MS) has been proven as an effective and desirable tool to explore and profile chemical constituents from complex materials such as natural products. A comprehensive chemical profiling strategy was established in our previous studies based on the combination of LC Q-exactive™ Orbitrap MS with metabolomics analysis, which has been successfully applied to global analysis for a variety of natural resources such as *Citrus reticulata* (Chachi) (Zhang et al., 2020a), *Citrus medica* (Zhang et al., 2022)and Ganpu tea (Xu et al., 2021). In the present study, we collected four representative cultivars of Chinese peonies that grow in the same experimental field under the same conditions. The global chemical identification was carried out firstly by LC-Q Orbitrap MS based on our established LC-HR MS identification strategy (Qiu et al., 2013). Then a validated quantitative and semi-quantitative analysis of multiple targets in CPF combined with untargeted and targeted metabolomics method was used to further compare the variations of different cultivars and different parts of CPF. Moreover, the correlation analysis between the chemical indexes and the anti-inflammatory/anti-oxidative activities was performed to indicate potential components that are responsible for the bioactivities. The present study could be a great instance in terms of the valorization of by-products as a new functional food from Chinese herb.

## 2 Materials and methods

### 2.1 Chemicals and reagents

Liquid chromatography (LC)-grade methanol, acetonitrile, and formic acid were purchased from Merck (Darmstadt, Germany). Ultra-pure water (18.2MΩ·cm) was pre-pared using a Milli-Q system (Millipore, Bedford, MA, USA). Reference standards of kaempferol, quercetin, taxifolin, apigenin, naringenin, peonidin-3-O-glucoside, cya-nidin-3-O-rutinoside, malvidin-3-O-glucoside, petunidin-3-O-glucoside, cyanidin 3-glucoside, cyanidin 3-(6’’-malonylglucoside), pelargonidin-3-O-glucoside, del-phinidin-3-O-Glucoside were purchased from Wuhan Zbsci Co., Ltd (Wuhan, China). The purity of the above references was higher than 98% (HPLC).

Griess reagent, Total Antioxidant Capacity Assay Kits with the DPPH, ABTS, and FRAP methods were from (eyotime Biotechnology (Shanghai, China). Tumor necrosis factor-α (TNF-α) and interleukin-6 (IL-6) by enzyme-linked immunosorbent assay (ELISA) kits were bought from Boster Biological Technology (Wuhan, China). Total Antioxidant Capacity Assay Kit for ORAC method was from Congyi Bio (Shanghai, China). All other reagents used were of analytical grade.

### 2.2 Plant materials

**Figure 1:**
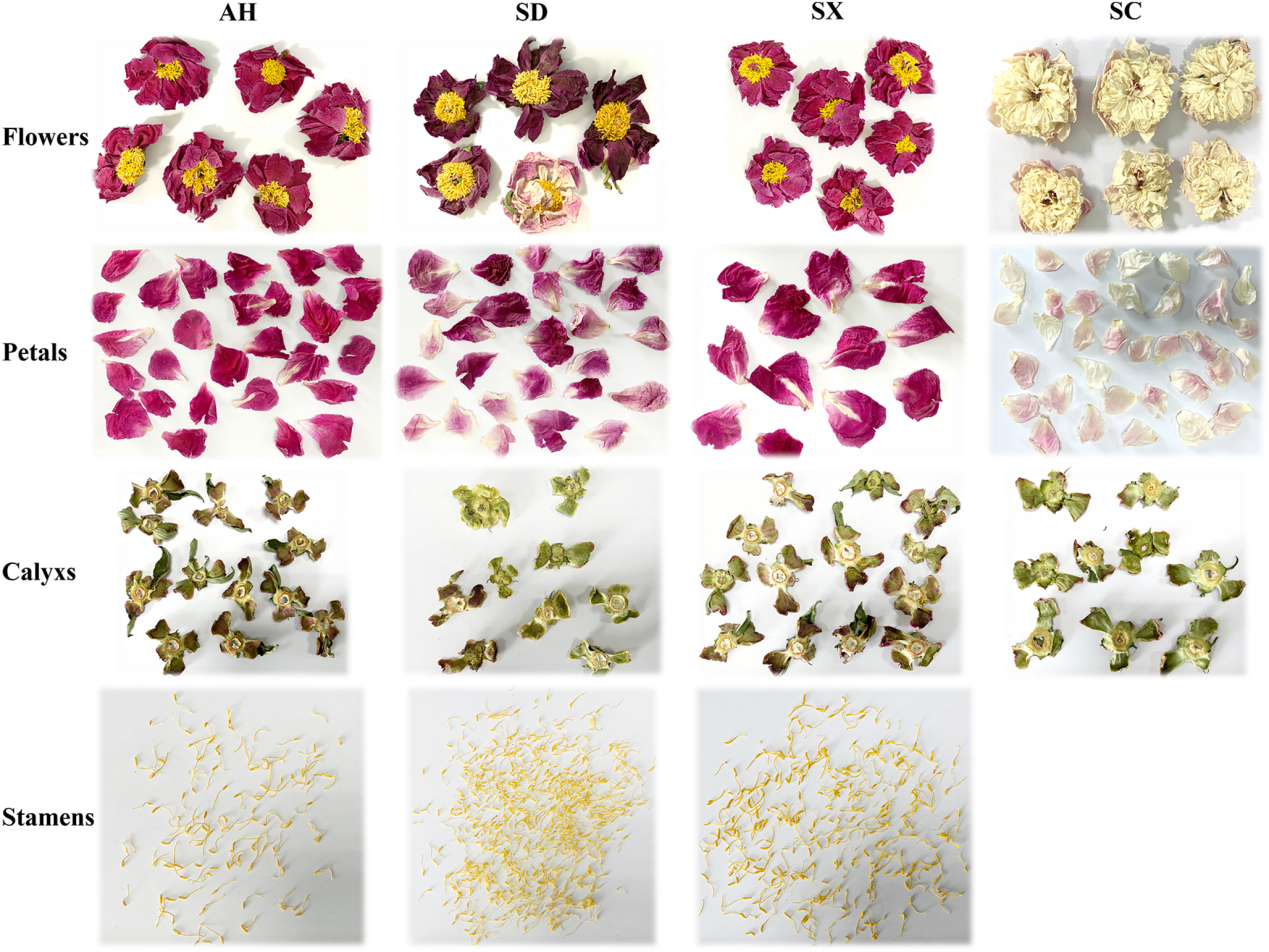
Morphological characteristics of four kinds of herbaceous peony flower samples.

Four cultivars of CPF from Anhui (AH), Sichuan (SC), Shandong (SD), and Shanxi (SX), were grown in separate areas of the same experimental field in Hezhe County, Shangdong province of China for five years. The cultivars names are 1# Xinshaofeng (AH), Guifeichacui (AH), Dafugui (SD), and Shuangchonglou (SX), respectively. The CPF samples were harvested at the same time in April 2021 when the flowers were in full bloom. The plants were authenticated by Prof. G.X., Zhou (Pharmacognosy Department, Jinnan University, Guangzhou, China), and a voucher specimen (2021SYH01) was stored at the Guangdong Provincial Academy of Chinese Medical Sciences. Samples were separated into three parts: stamens, petals, and calyx (**Figure 1**), except SC cultivar, of which the stamens were not separated from the petals. Samples were placed in a freeze-dryer at -80°C for 48 hours and then grounded, sealed, and stored at 4°C before use.

### 2.3 Preparation of sample solutions

0.1 g of each powder sample was accurately weighed and then extracted with 3 mL methanol-water (70:30, v/v) containing 0.1% hydrochloric acid for 24 hours at room temperature. The extract was centrifuged at 5,000 rpm for 15 minutes. The supernatants were collected and filtered through a 0.22 μm filter (JinTeng, Tianjin, China). Quality control (QC) sample was made by assembling the same amount of each sample solution. In the LC-MS run, samples were analyzed in random order and a QC sample was inserted in every six testing samples to monitor the reproducibility and stability of the method.

### 2.4 UHPLC-Q-Orbitrap MS analysis

LC-MS experiments were performed on a U3000 UHPLC (Thermo Fisher Scientific, Waltham, MA, USA) coupled with a Q-Exactive Orbitrap hybrid MS system (Thermo Fisher Scientific, Rockford, IL, USA). The chromatographic separation was performed on a Waters HSS T3 column at a flow rate of 200 µL/min. The mobile phase consisted of water (A) and acetonitrile (B), both containing 0.1% formic acid. The elution gradient was set as follows: 90% A (0-1 min), 90-82% (1-3 min), 82-68% A (3-6 min), 68-54% A (6-8.5 min), 54-50% A (8.5-10 min), 50-10% A (10-13 min), 10% A (13-15min), 10-90% A (15-17min), 90% A (17-19min). The column temperature was 30°C and the injection volume was 2 µL.

The MS data were acquired using an Electron Spray Ionization (ESI) source both in negative and positive modes. The parameters were as follows: Ion spray voltage, 3,500 V in positive mode and 3,700 V in negative mode; capillary temperature, 350 °C; aux gas, 15 arb; sheath gas, 40 arb; MS resolutions for survey scanning and data-dependent acquisition were 35,000 and 17,500, respectively; scan range, m/z 100 ~ 1,200; and the normalized collision energy for DDA, 35 eV. Xcalibur software (version 3.1, Thermo Fisher Scientific, Waltham, MA, USA) was used for data acquisition.

### 2.5 Qualitative analysis

The identification procedure of the chemical components of CPF was summarized as the following three levels of strategy. Level one, components are directly and accurately identified by comparing the retention time, quasi-molecular and fragment ions with reference standards analyzed under the same condition. Level two, components were identified by comparing their orthogonal MS features (precursor ion and characteristic fragment ions) to those known compounds from *P. lactiflora* and related species. Relative retention time would be considered as well. Level three, identification was conducted based on the fragmentation patterns and diagnostic fragment ions and/or neutral losses patterns summarized from the analysis of standards and known compounds. For these unknown compounds, highly accurate diagnostic fragment ion filtering and characteristic neutral loss filtering strategies were used to classify them into specific chemical subfamilies. In most cases, two or more common MS/MS product ions of the components with the same core substructures were selected as the characteristic fragment ions. Typical characteristic neutral losses of moieties such as C_2_H_2_O (acetyl), C_7_H_6_O_5_ (galloyl), C_3_H_2_O_3_ (malonyl), C_6_H_10_O_5_ (hexosyl, hex), and their combinations, which can be deduced from the common fragmentation behaviors of the same type, were used to characterize these derivatives of known compounds.

### 2.6 Quantitative analysis

Among all identified compounds, 69 compounds with high peak intensities, as well as good peak shapes and resolution were selected for quantitative analysis. The methodology was evaluated firstly by testing the linearity, limit of detection (LOD), limit of quantification (LOQ), and inter-day and intra-day precision of the 14 standard compounds. The calibration curves were obtained by plotting the peak area versus concentration, while the LOD and LOQ were determined by signal-to-noise ratios greater than 3 and 10. The intra-day and inter-day precision were evaluated by preparing three different concentration levels (low, medium, and high) of reference samples in one day and re-peated for three consecutive days, respectively. The Intermediate precision was tested using medium reference samples by two experimenters at the same lab. The recovery rates of the fourteen analytes were determined at one level and calculated % recovery of known amounts of added to samples.

The results were expressed as relative standard deviation (RSD%). Since only 14 references were used for quantification, the quantities of these 14 components were calculated by calibration curves directly, while the rest of the components were quantified by using standard curves of those references that shared similar core structures with the target components. For example, the quantification of quercetin glucoside was determined by the standard curve of quercetin; the quantification of centaureidin di-glucoside was determined by the standard curve of centaureidin glucoside.

### 2.7 Evaluation of antioxidant activity

1,1-Diphenyl-2-picrylhydrazyl (DPPH) radical scavenging activity was assayed by the Total Antioxidant Capacity Assay Kit with the DPPH method 30μL calibration solution (Trolox), sample or blank (70% methanol) was added to 270 μL of DPPH (6×10-2 mM in 80% methanol) in different wells of a 96-well microplate, which was instantly kept in the dark for 30 min. The absorbance of the reaction mixture was then measured at 517 nm. The standard curve was established using Trolox ranging from 0.02 to 0.24 mM. The results were calculated from the linear calibration curve and expressed as μM Trolox equivalent (TE)/g dry weight of the sample.

2,2′-Azinobis-(3-ethylbenzthiazolin-6-sulfonic acid) (ABTS) radical cation scavenging activity was assayed by the Total Antioxidant Capacity Assay Kit with ABTS method. A stock solution was made by mixing the ABTS solution and oxidizing agent in equal volumes and kept in the dark for 16 hours. Then it was diluted in 80% methanol to obtain a working solution with absorbance of 0.7 ± 0.05 at 734 nm. 10 μL calibration solution (Trolox), sample or blank solution (80% methanol) were added to 200 μL of work solution in a 96-well microplate, which was incubated in the dark for 10 min. The absorbance of the reaction mixture was then measured at 734 nm. The standard curve was established using Trolox ranging from 0.1 to 0.9 mM. The results were calculated from the linear calibration curve and expressed as μM Trolox equivalent (TE)/g dry weight of the sample.

Ferric ion-reducing antioxidant power (FRAP) was assayed by the Total Antioxidant Capacity Assay Kit with the FRAP method. A working solution was prepared by mixing the tripyridyltriazine (TPTZ) solution, TPTZ dilution, and detective buffer at a ratio of 10:1:1 (v/v). The working solution was warmed in a water bath at 37°C and should be used within 2 hours. 5 μL calibration solution (Trolox), sample, or blank (70% methanol) was added to 180 μL of work solution in different wells of a 96-well microplate, then the mixture was incubated in the dark for 6 min at 37°C. The absorbance of the reaction mixture was then measured at 593 nm. The standard curve was established using FeSO_4_ solution ranging from 0.15 to 1.5 mM. Results were calculated from the linear curve.

Oxygen radical absorbance capacity (ORAC) was tested by the Total Antioxidant Capacity Assay Kit with the ORAC method. The assay was conducted in a 96-well microplate with black opaque. Reagent 1 working solution was prepared by adding 10 mL dilution to 100 μL reagent 1. Reagent 2 working solution was prepared by adding 15 mL dilution to reagent 2. Briefly, 50 μL of calibration solution (Trolox), sample, or blank solution were added to 50 μL of reagent 1 working solution in each well of the microplate and the mixture was kept for 30 min at 37°C in the dark, then 100 μL of reagent 2 working solution was added immediately, and the microplate was placed into a micro-plate reader with the parameters set as follows: shaking time, 5 s; incubation temperature, 37°C; excitation wavelength, 485 nm; excitation wavelength, 520 nm. The fluorescence was read every 2 min for 90 min. The standard curve was established using Trolox ranging from 2.5 to 60 μM. The final ORAC results were calculated based on the differences in areas under the fluorescence quenching curve (AUC) between a sample and blank and expressed as μM Trolox equivalent (TE)/g dry weight of the sample. The AUC was calculated by Eq.


(1) $$AUC=\frac{f_{0}}{f_{0}}+\frac{f_{1}}{f_{0}}+\frac{f_{2}}{f_{0}}+\ldots +\frac{f_{n-1}}{f_{0}}+\frac{f_{n}}{f_{0}}$$

where f_0_ indicated initial fluorescence reading at 0 min and fn indicated fluorescence reading at the nth detection.

### 2.8 Anti-inflammatory activity assay

RAW264.7 cells (2 × 10^5^ cells/well) were seeded into 24-well plates and induced by 1 μg/mL LPS, with or without CPF for 24 h. Then the cell supernatant was collected and the content of NO was determined with Griess reagent at 540 nm with a microplate reader. Meanwhile, the supernatant was used to measure the levels of proinflammatory cytokines including tumor necrosis factor-α (TNF-α) and interleukin-6 (IL-6) by enzyme-linked immunosorbent assay (ELISA) kits in strict accordance with the manufacturers’ instructions.

### 2.9 Determination of total phenolic, flavonoid, and anthocyanin contents

The total phenolic contents (TPC) of different parts of CPF were measured according to the previous protocol (Duan et al., 2006). Briefly, 0.5 mL of 0.2 N Folin-Ciocalteu reagent was added to 0.1 mL of sample extract solution. After 5 min of incubation, 0.4 mL of 7.5% Na_2_CO_3_ solution was sequentially added, followed by a two-hour incubation in the dark. The absorbance of each mixture was read at 760 nm in a microplate reader. Gallic acid solutions with a series of concentrations were measured at the same condition for drawing the regression curve. The TFC result was expressed as gram gallic acid equivalents (GE)/100 g sample in dry weight.

The total flavonoid contents (TFC) of different parts of CPF were measured according to the previous protocol with slight modifications (Bai et al., 2021). In brief, 0.5 mL of sample extract solution was added with 0.15 mL of 5% NaNO_2_ solution and 0.15 mL of Al (NO_3_)_3_ solution (0.3 M). After incubation for 6 min, 2 mL of NaOH solution (4%, w/v) was sequentially added and the absorbance of the mixture was read at 510 nm. Series concentrations of rutin solutions were measured at the same condition for drawing the regression curve. The TFC result of each sample was expressed as gram rutin equivalents (RE)/100 g sample in dry weight.

Total anthocyanins content (TAC) was determined using a modification of the pH differential method (Moldovan et al., 2016). Aqueous buffer solutions at pH 1 and 4.5 were prepared from 0.025 M potassium chloride/hydrochloric acid buffer and 0.4 M sodium acetate/acetic acid buffer, respectively. Each sample (300 µL) was mixed with 2.7 mL of buffer. After equilibrating at room temperature in the darkness for 15 min, the absorbance at 515 and 700 nm was read using a UV-visible spectrophotometer and extraction solvent as the blank. The total anthocyanins content was calculated as equivalents of cyanidin-3-glucoside per gram of CPF sample using the previous equation (Moldovan et al., 2016).

### 2.10 Data preprocessing and statistical analysis

Data acquisition was achieved by Xcalibur software (version 3.1, Thermo Fisher, Waltham, MA, U.S.A.). The quantitative data were processed with the LC-Quan module (Thermo Fisher, Waltham, MA, U.S.A.). For untargeted metabolomics analysis, raw LC-MS data were processed by Compound Discovery, version 3.2, including feature detection, alignment, and normalization. The mass window for feature detection and alignment was set at 5 ppm. The retention time (RT) window was set at 0.2 min. The normalized data matrix was exported into SIMCA-P 14.1 (Umetrics, Sweden) for principal component analysis (PCA). The established models were assessed by calculating the R2 and Q2 values, which represented the goodness and predictability of the models, respectively. A one-way analysis of variance (ANOVA) statistical test was performed to analyze statistical significance between groups using SPSS software (version 25.0). Heat map analysis was conducted by R-Studio and Metware Cloud (https://cloud.metware.cn/) to display the quantitative changes in the chemical constituents.

For antioxidant activity, total phenolic, total flavonoid, and anthocyanin contents assays, all the samples were tested in triplicates. Statistical analysis was conducted by using the one-way analysis of variance and *p*<0.05 was considered to be significant.

## 3 Results

**Figure 2:**
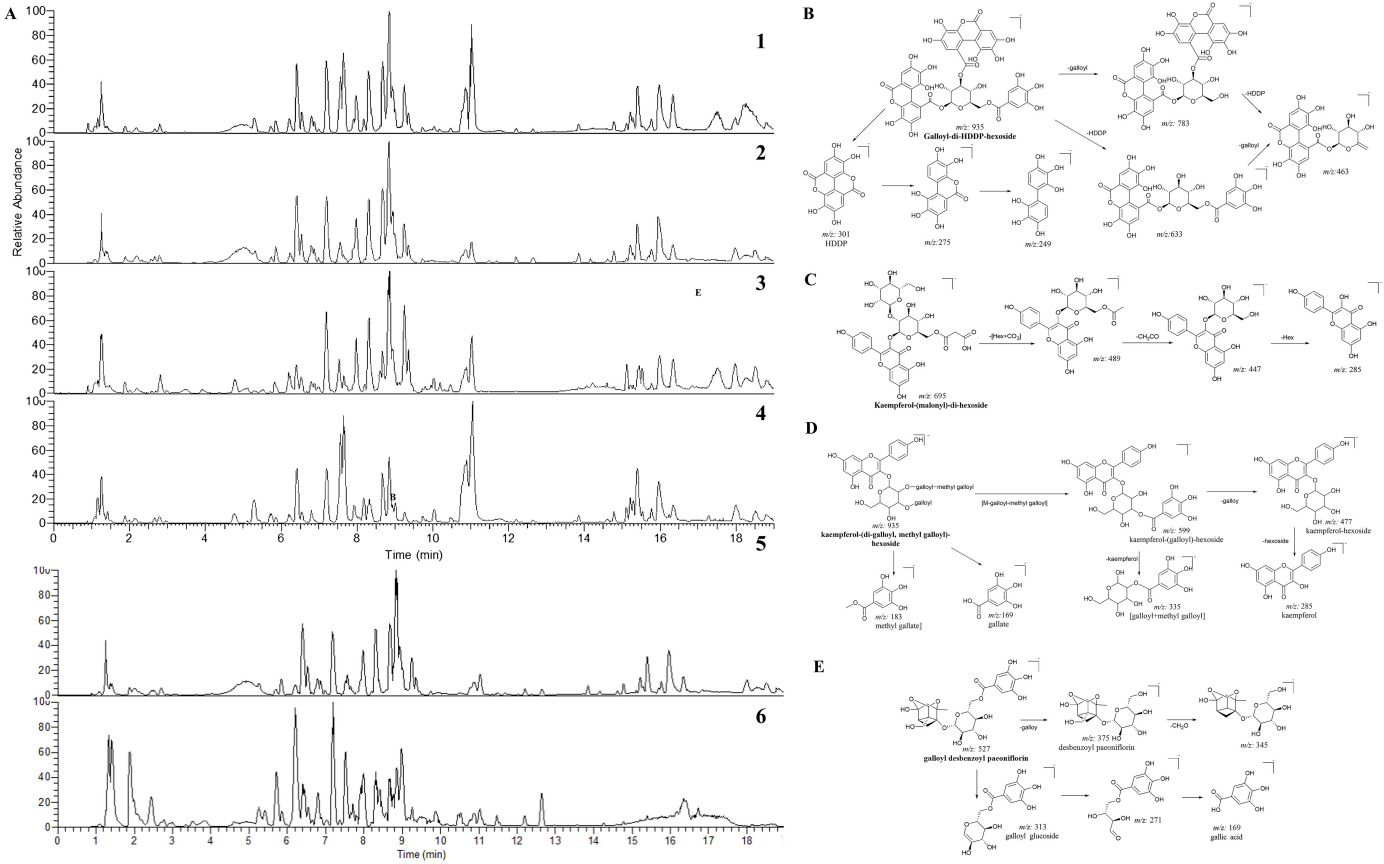
The LC-MS chromatograms of CPF and the fragmentation pathways of selected compounds. **(A)** The base peak chromatograms (BPC) of whole flower (A1), petals (A2), calyx (A3) and stamens (A4); The BPC of petals in positive (A5) and negative (A6) ion modes of mass spectrometry. The proposed fragmentation pathways of galloyl-di-HDDP-hexoside **(B)**, kaempferol-(malonyl)-di-hexoside **(C)**, kaempferol-(di-galloyl, methyl galloyl)-hexoside **(D)**, and galloyl desbenzoyl paeoniflorin **(E)**.

The typical base peak chromatograms (BPC) of each part are illustrated in **Figure 2**, where the disparity in the relative peak height and peak area can be drawn by visual in-spection. In order to obtain detailed knowledge concerning the changes of individual chemical constituents, a systematic chemical investigation was conducted first.

### 3.1 Identification of chemical components

According to the qualitative Analysis strategy, 150 components were characterized, of which the retention times, molecular formulae, and the high-resolution precursor and characteristic fragment ions are listed in **Supplementary Table S3**. Except those major and representative components, more than 50 compounds were detected from *Paeonia lactiflora* for the first time. In addition, the fragmentation behaviors and characteristic fragments of those new detected components were elaborated.

#### 3.1.1 Identification of hydrolysable tannins

Previous reports showed that the fruit of *Paeonia lactiflora* possess considerable number of tannins (Li et al., 2021). Hydrolysable tannins are an important group of secondary metabolites that exhibit anti-angiogenic, antioxidant, anti-inflammatory, and anti-ulcerative properties. For the first time, the present LC-MS study indicated that CPF is rich in hydrolysable tannins but not condensed tannin. Herein thirty-one compounds were rapidly characterized as hydrolysable tannins based on their characteristic fragment ions in Orbitrap MS.

Simple gallotannins included di-gallic acid, tri-gallic acid, tetra-gallic acid and their isomers. They all showed common characteristic ions at *m/z* 169.0131 and 125.0230, referring to [gallic acid] and [gallic acid - CO_2_] residues, respectively. Compound 18 exhibited a precursor ion at *m/z* 183.0298 [M-H]^-^ ion, yielding two odd-electron fragments at *m/z* 168.0052 [M –·CH_3_] and 124.0152 [M – CO_2_ –·CH_3_]^-^, indicating a methyl gallic acid (Qiu et al., 2022). In addition, compounds 25 and 31, with their [M–H]^–^ ion at *m/z* 335.0408 [M–H]^–^ ion and main fragments at *m/z* 183, 168, and 124, were identified as methyl di-gallate. Another common type of gallic acid derivatives found in CPF were galloyl hexosides. Those precursor ions showed fragments of *m/z* 169 [M−H−162 Da]^–^ and 125 [M−H−162 Da−44 Da]^–^, corresponding to the characteristic neutral loss of a hexoside unit. Accordingly, these two characteristic fragments facilitated the identification of galloyl hexoside analogs such as di-galloyl hexoside, tri-galloyl hexoside, tetra-galloyl hexoside, and penta-galloyl hexoside.

Compounds 26 and 30 were identified from Chinese peony for the first time. Notably, their common fragmentation behaviors on Orbitrap MS were characterized by characteristic fragment ions due to successive losses of C_7_H_4_O_4_ and C_7_H_6_O_5_ units, indicating they were gallotannins. Take compound 26 as an example. It produced [M−H]^–^ ion at *m/z* 801.1159 (C_35_H_30_O_22_), and further fragmented into *m/z* 649.1077 (C_28_H_25_O_18_), 631.0933 (C_28_H_23_O_17_), 479.0836 (C_21_H_19_O_13_), and 327.0718(C_14_H_15_O_9_), representing the neutral losses of [C_7_H_4_O_4_], [C_7_H_6_O_5_], [C_7_H_6_O + C_7_H_4_O_4_], and [C_7_H_6_O + 2×C_7_H_4_O_4_], respectively. The final fragment product ion at *m/z* 193.0133 represented methyl glucoside, thus, compound 26 was deduced to be methyl-tetra-galloyl hexoside (**Supplementary Figure S1**).

Ellagitannins and its major components ellagic acid present in some fruits and seeds. Their structures are characterized by the distinct hexahydroxydiphenol (HHDP) group. Herein, we reported five HHDP hexosides from CPF for the first time. Peak 21 displayed [M−H]^–^ ion at *m/z* 935.0800 and produced major fragments at *m/z* 300.9988, 275.0199, 249.0400, 169.0129, indicating a hexahydroxydiphenol (HHDP) group (Qiu et al., 2022). Other fragments such as 783.0720, 633.0732, and 463.0501 referred to the losses of galloyl, HHDP, and [galloyl+HHDP] units, respectively (**Figure 2B**). Thus, it was tentatively identified as galloyl-di-HDDP-hexoside. By using the fragment ions filtering of 300.9988, 275.0199, 249.0400, several HDDP-hexoside (7, 10, 15, and 19) derivatives were also detected.

#### 3.1.2 Identification of flavonoids

A total of 70 compounds were identified as flavonoid derivatives. Five flavonoid aglycones, taxifolin, quercetin, apigenin, naringenin, and kaempferol, were unambiguously identified by comparing their retention times and mass spectrometry features with those standard compounds. As described in previous studies (Bai et al., 2021), the MS fragmentation of flavone/flavanone *O*-glycosides in both positive and negative ion modes is featured by producing the prominent base fragment ion of [M-glycosides]. Generally, neutral losses of these glycosides refer to rutinoside (308 Da), hexoside (162 Da), deoxyhexoside (146 Da), and pentoside (132 Da).

Notably, a series of modified flavonoid glycosides, where the glycoside residue is acylated with aromatic or aliphatic acids (malonic, gallic, and acetic acids, to name a few), were identified. For instance, several malonyl-substituted flavonoid glycosides, including isorhamnetin-malonyl dihexoside, isorhamnetin-malonylhexoside, kaempferol-malonyl dihexoside, kaempferol-malonyl hexoside, and so on were reported. Those malonyl derivatives showed [F+C_3_H_2_O_3_ (86 Da)] precursor ions (herein, F denotes the corresponding flavonoid glycoside) in the survey scan. Specifically, when it was modified by malonyl on the glycoside part, prominent fragment ions referring to neutral losses of malonyl residue (86 Da, CHO) and/or malonyl glucoside residue (162 + 86 Da) would present in MS/MS spectra, while other fragment ions were identical to those prototypical compounds. Take kaempferol-(malonyl)-di-hexoside as an example. It produced precursor ion at *m/z* 695.1464, which further fragmented into the base fragment peak at *m/z* 447.0911 [M-(162 + 86 Da)-H]. In addition, another characteristic fragmentation ion at *m/z* 489.1005 was resulted from the cleavage of the malonyl group, which shows neutral loss of [162(glu)+44 Da(CO_2_)]. The proposed fragmentation pattern is illustrated in **Figure 2C**. As natural derivatives, malonyl glycosides were reported from many plant species and their content may be associated with antioxidant capacities (Kwak et al., 2007). To our knowledge, this is the first time for the detection of malonyl-substituted flavonoids in CPF.

Likewise, acetyl, gallic acyl, and *p*-coumaroyl-substituted flavonoid glycosides were also detected and characterized by applying the same strategy (**Supplementary Figure S1**). In general, they showed the characteristic precursor ions such as [F+C_2_H_2_O (42 Da)], [F+C_7_H_4_O_4_ (152 Da)] or [F+C_9_H_6_O_2_ (146 Da)] in survey scans, respectively, and exhibit the same prominent aglycone ions in their MS/MS spectra with their prototype components.

For compound 77, it showed a [M-H]^–^ at *m/z* 935.1528 (C_43_H_36_O_24_), which further produced as same fragments at *m/z* 599.1040, 447.0919, 313.0559, 285.0400, 169.0129, 151.0028 as kaempferol-(galloyl)-hexoside, indicating it was kaempferol-(galloyl)-hexoside derivative. The base fragment ion at *m/z* 183.0289 was deduced to be a methyl gallate module. Both the fragment at *m/z* 335.0399 and the neutral loss for *m/z* 599.1040 further indicated the [methyl gallate + gallate] unit. The fragmentation pathway is illustrated in **Figure 2D**. Thus it was tentatively identified as kaempferol-(di-galloyl, methyl galloyl)-hexoside, which is a potential new component.

#### 3.1.3 Identification of anthocyanins

Anthocyanins are natural pigments belonging to the flavonoid group. So far, more than 25 natural anthocyanins have been identified, of which 95% are derived from six aglycone porotypes, namely cyanidin (Cy), paeonidin (Pn), pelargonidine (Pg), malvidin (Mv), delphinidin (Dp), and petunidin (Pt) (Chen et al., 2022). In the present study, 16 compounds were identified as anthocyanins. Cy-diglu, Pn-diglu, Dp-glu, Cy-glu, Pt-glu, Cy-rutin, Pg-glu, Pn-glu, Mv-glu, Cy-malglu, Dp, Cy, Pt, Pg, and Mv were unambiguously identified by comparison the retention time and mass spectrometric information with the standard compounds. Other anthocyanins were tentatively identified based on level 3 strategy.

#### 3.1.4 Identification of monoterpene glycosides

Pinane monoterpene glycosides are the characteristic components of *P. lactiflora*. Two prominent peaks (peaks 125 and 127) were identified as albiflorin and paeoniflorin, according to their distinctive fragmentation patterns (Nie et al., 2021). As the type of benzoate ester of monoterpene lactone glycosides, their typical characteristic fragment ions in negative mode are at [M- CH_2_O (30 Da)], [[M-CH_2_O-benzoate], *m/z* 327.1077[M-2× CH_2_O-benzoate], and *m/z* 121.0280[benzoyl-H] (**Supplementary Figure S1**).

Compounds 119, 120, 122 - 128, 130, and 131 were determined to share the same pinane monoterpene skeleton as paeoniflorin. In general, these compounds can also form a characteristic fragment ion of [M-H-CH_2_O]^-^, as well as fragments referring to the loss of the benzoyl group. For instance, compound 124 generated characteristic fragment ions of *m/z* 489.1611 [M-H-CH_2_O- benzoate]^-^ and *m/z* 121.0281 [benzoate -H]^-^, and a characteristic pinane monoterpene (C_10_H_9_O_3_) fragment ion at *m/z* 177.0546. Thus, it was deduced to be albiflorin hexoside.

Herein two potential new monoterpene glycosides were tentatively identified. Compound 135 and 136 showed their [M-H]^-^ ion at *m/z* 527.1409, producing fragment ion at *m/z* 375.1293 due to the neutral loss of galloyl (152 Da), which further loss 30 Da [CH_2_O] to produce characteristic ion at *m/z* 345.1187. The rest characteristic fragments such as 313.0565, 271.0462, and 169.0132 are the same as galloylpaeoniflorin (130). Based on this information, they were tentatively identified as galloyl desbenzoyl paeoniflorin or galloyl desbenzoyl albiflorin. The fragmentation pathway was proposed in **Figure 2E**.

#### 3.1.5 Identification of other phenolics

Fourteen compounds in the samples were tentatively identified as phenolic acids. To our knowledge, 15 of these compounds were identified for the first time from this genus. In this study, we identified protocatechuic acid hexoside (137,139), *p*-hydroxybenzoic acid hexoside (138), *p*-coumaryl glucarate (140,142,143), benzoic acid glucarate (141,144,145,147) and their isomers. They showed the characteristic fragment ions in their product ion spectra by elimination of hexoside (162Da) and glucarate (192 Da) moieties.

### 3.2 Metabolomics profiling analysis of CPF from different parts and cultivars

**Figure 3:**
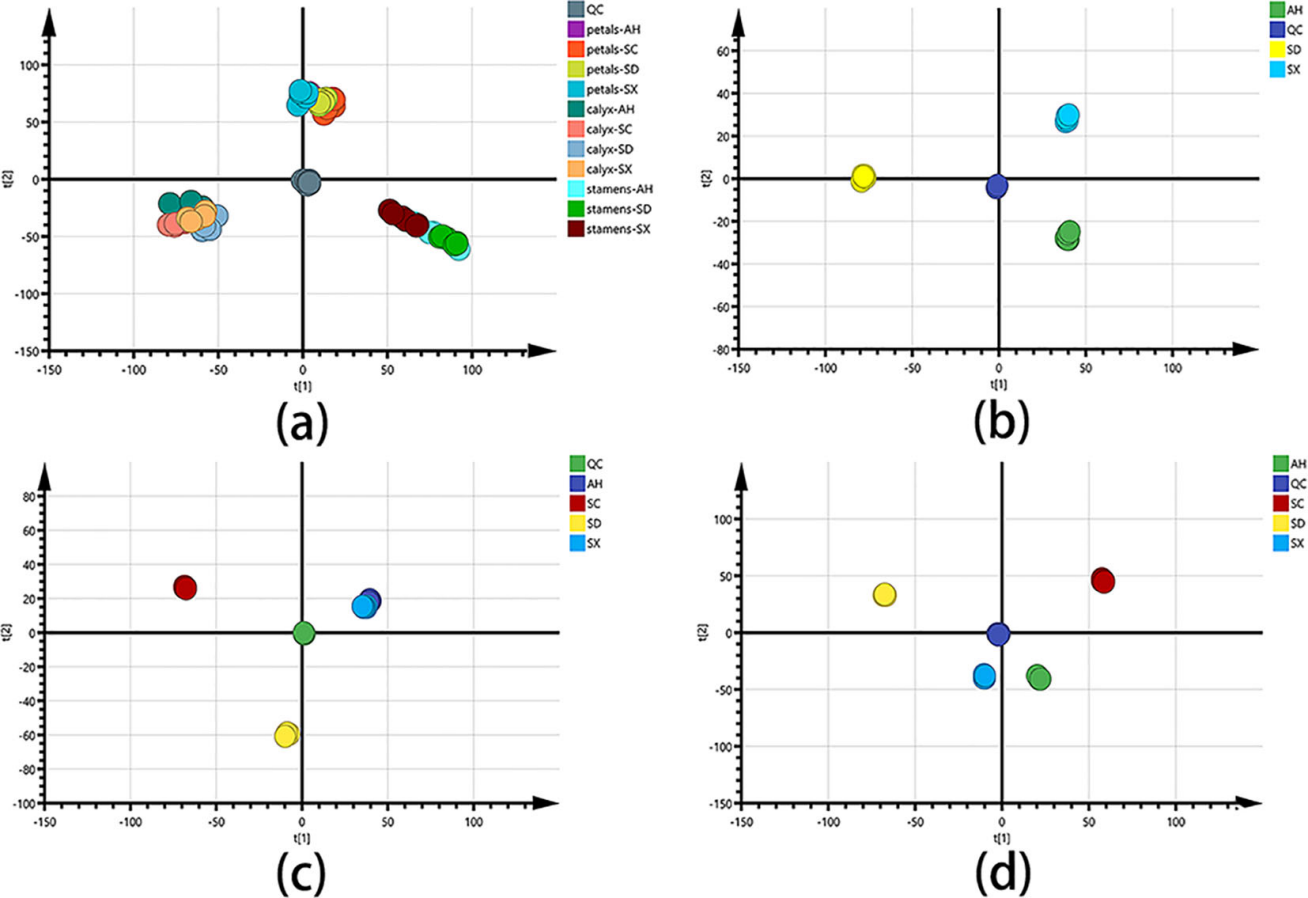
PCA score charts for different group settings: **(A)** PCA score plot of all CPF samples (n = 6), with R^2^X and Q^2^ of 0.836 and 0.747, respectively. **(B)** PCA score plot of stamen samples. **(C)** PCA score plot of petal samples. **(D)** PCA score plot of calyx samples. SC, AH, SD, and SX denote cultivars from Sichuan, Anhui, Shandong, and Shanxi, respectively.

**Figure 4:**
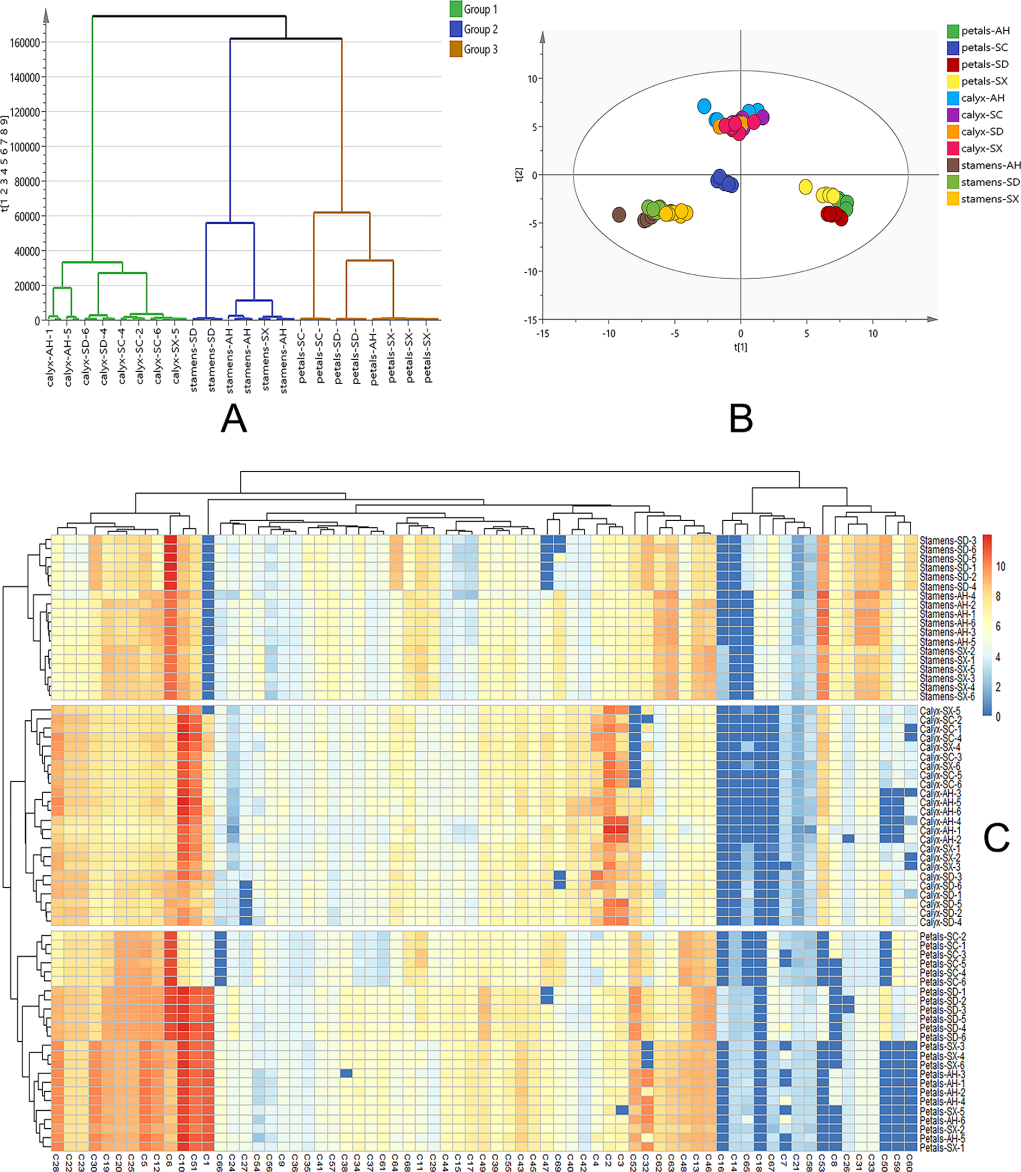
**(A)** Clustering analysis of all the CPF samples. Group 1 in green represents 24 calyx samples, group 2 in blue represents 18 stamen samples and group 3 in brown represents 24 petal samples. **(B)** Targeted PCA analysis of 69 quantification data. R^2^X = 0.901, Q^2^ = 0.830. **(C)** Heatmap constructed from quantification data. The abscissa represents the quantified components and the ordinate represents samples. SC, AH, SD, and SX denote cultivars from Sichuan, Anhui, Shandong, and Shanxi, respectively.

To capture an overall picture of the primary distinction of CPF in different parts and cultivars, an untargeted PCA and Hierarchical Cluster Analysis (HCA) models were created firstly. The clear chemical variation between three flower parts could be seen from the PCA score plot (**Figure 3A**) and HCA plot (**Figure 4A**) of all the 66 samples, while the QC group gathered together at the origin of the orthogonal coordinate. Samples from the same plant part were selected to further PCA analysis (**Figures 3B–D**). They explicitly showed the distinct chemical compositions among those cultivars even though they were grown at the same place. Previous studies indicated (Li et al., 2023; Yang et al., 2023) significant chemical differences between Chinese Peony cultivars. Although both genetic and environmental factors can affect the chemical phenotype, our result showed that the chemical distinctions between those cultivars could be solely attributed to the intrinsic cause.

### 3.3 Quantitative comparison and multivariate analysis

Subsequently, targeted multivariate analysis and comparison between those samples, based on quantifying the contents of individual compounds in CPF, were constructed to get a more precise picture of chemical differences. Previously, we set up a simi-quantification data-based targeted metabolomics strategy, which had been successfully applied for fast analysis and comparison of plant-derived food resources, such as tangerine (Zhang et al., 2020a), Ganpu tea (Xu et al., 2021) and citron (Zhang et al., 2022). Herein, we used similar strategy to address the CPF samples from different flower parts and cultivars.

Firstly, 14 reference compounds, covering anthocyanins and flavonoids, were used to construct and validate the quantitative methodology. The selection of the 14 quantitative targets is based on the content in CPF implied by previous chemical investigation (Zhang et al., 2020b; Liu et al., 2023). Also, the commercial availability of those reference standards was considered. Validation of the quantification method in terms of linearity, LOD, LOQ, intra-day, inter-day precision, Intermediate precision and recovery rate was conducted by using 14 standards, which covered the main anthocyanins, flavonoids, and monoterpenoids in CPF. As shown in **Supplementary Table S1**, all 14 standards showed good linearity between concentrations and corresponding MS peak areas with correlation coefficients >0.995. The intra-day and inter-day of most standards at low, medium, and high concentration levels were<10%, and the Intermediate precision RSDs were less than 12%, indicating acceptable precision of the method. The recovery rates of 14 analytes were between 85.71% to 112.09% with RSDs less than 10.62%. The LODs and LOQs for 14 analytes were between 0.2–1.0 and 0.4– 4.0 ng/mL, respectively, suggesting good sensitivity of the method. Other than the 14 reference compounds, components with high peak intensities (above 1 × 10^6^ in the QC sample) and good separation were selected for further quantitative comparison. Those targets would be quantified by using the standard curve of reference compounds from the same structure prototype. Thus, a total of 69 targets were selected and they were quantified or semi-quantified (**Table 1**; **Supplementary Table S2**).

**Table 1:** The contents or relative contents of 69 targets quantified by LC-MS.

No.	compounds	petal-AH	petal-SC	petal-SD	petal-SX	petal	calyx-AH	calyx-SC	calyx-SD	calyx-SX	calyx	stamen-AH	stamen-SD	stamen-SX	stamen
C1	Cyanidin-3,5-O-diglucoside	2016.09 ± 171.93	22.21 ± 4.3	2019.61 ± 170.59	1767.45 ± 117.59	1456.34	168.8 ± 62.47	214.92 ± 95.46	261.57 ± 115.32	180.71 ± 123.4	206.50	N.D.	N.D.	N.D.	N.D.
C2*	Delphinidol-3-O-glucoside	82.18 ± 24.19	62.18 ± 3.05	97.05 ± 33.65	143.59 ± 24.93	96.25	1530.61 ± 944.34	1132.72 ± 246.66	843.15 ± 329.47	1017.1 ± 295.26	1130.90	62.85 ± 10.75	91.57 ± 18.24	56.46 ± 4.48	70.29
C3	Delphinidin	108.55 ± 24.3	61.02 ± 17.87	162.19 ± 13.11	95.23 ± 48.39	106.74	1171.81 ± 1121.39	586.7 ± 332.59	708.5 ± 553.06	492.14 ± 318.12	739.79	81.42 ± 9.48	84.28 ± 11.43	87.82 ± 9.54	84.51
C4*	Cyanidin-3-O-glucoside	55.44 ± 40.11	31.55 ± 23.87	65.34 ± 19.55	42.96 ± 3.79	48.82	342.37 ± 194.14	466.18 ± 258.9	508.72 ± 447.06	357.74 ± 185.43	418.75	82.23 ± 21	46.6 ± 7.31	46.39 ± 14.39	58.41
C5	Cyanidin	1251.17 ± 46.79	635.9 ± 91.87	720.82 ± 11.89	1131.46 ± 93.4	934.84	185.29 ± 74.06	223.89 ± 53.86	228.5 ± 61.76	198.38 ± 34.12	209.01	381.32 ± 61.18	342.26 ± 29.4	249.42 ± 36.08	324.33
C6*	Petunidin-3-O-glucoside	364.34 ± 17.92	2532.6 ± 285.86	2412.18 ± 104.34	512.43 ± 52.32	1455.39	169.87 ± 25.04	217.22 ± 29.5	532.78 ± 68.83	172.9 ± 76.73	273.19	1573.36 ± 58.9	3634.04 ± 224.8	1478.57 ± 76.86	2228.66
C7* C8*	Cyanidin-3-O-rutinoside	16.48 ± 22.1	4.83 ± 3.77	31.32 ± 19.5	8.82 ± 12.41	15.36	8.15 ± 0.59	8.48 ± 1.46	7.7 ± 1.12	6.55 ± 3.29	7.72	13.48 ± 0.74	14.23 ± 1.94	12.12 ± 1.25	13.28
	Pelargonidin-3-O-glucoside	17.92 ± 13.9	35.88 ± 40.14	N.D.	N.D.	13.45	40.55 ± 18.76	54.72 ± 5.04	51.07 ± 3.39	52.28 ± 2.32	49.65	83.31 ± 9.36	66.28 ± 2.95	115.46 ± 12.18	88.35
C9	Pelargonidin	25.59 ± 3.95	17.4 ± 1.25	20.27 ± 2.11	23.99 ± 2.96	21.81	40.36 ± 12.84	34.61 ± 6.68	29.58 ± 3.05	26.65 ± 4.37	32.80	17.15 ± 5.21	23.21 ± 3.43	15.84 ± 1.96	18.73
C10*	Peonidin-3-O-glucoside	2843.22 ± 286.11	155.8 ± 38.1	3221.15 ± 510.57	2783.51 ± 303.97	2250.92	2545.34 ± 813.06	2610.63 ± 780.38	903.46 ± 394.03	1912.69 ± 485.31	1993.03	528.84 ± 98.22	446.28 ± 65.22	492.73 ± 91.93	489.28
C11*	Malvidin-3-O-glucoside	28.72 ± 5.37	265.29 ± 87.03	123.94 ± 29.59	37.65 ± 10.7	113.90	29.68 ± 2.85	23.99 ± 2.53	39.26 ± 10.57	28.75 ± 3.12	30.42	212.58 ± 38.42	265 ± 28.05	285.66 ± 24.27	254.41
C12*	Cyanidin-3-(6”-malonylglucoside)	859.45 ± 46.09	383.67 ± 33.06	718.99 ± 38.73	731.46 ± 100.81	673.39	234.71 ± 101.99	269.17 ± 52.41	315.98 ± 72.21	264.18 ± 56.71	271.01	470.47 ± 31.75	89.93 ± 14.61	357.7 ± 22.06	306.03
C13	Kaempferol-dihexoside	522.02 ± 11.48	429.34 ± 17.21	557.16 ± 15.72	478.85 ± 23.05	496.84	63.51 ± 28.08	83.84 ± 15.13	104.31 ± 19.16	73.84 ± 13.12	81.37	335.35 ± 87.32	225.21 ± 33.87	429.18 ± 29.96	329.91
C14	Dihydrokaempferol-hexoside	8.18 ± 0.25	3.57 ± 0.12	5.62 ± 0.18	6.98 ± 0.54	6.09	N.D.	N.D.	N.D.	N.D.	N.D.	N.D.	N.D.	N.D.	N.D.
C15	Kaempferol-(malonyl)-dihexoside	94.89 ± 3.37	63.9 ± 4.45	117.44 ± 3.45	90.1 ± 8.79	91.58	25.57 ± 14.6	37.3 ± 8.65	39.21 ± 9.01	32.21 ± 7.64	33.57	16.64 ± 6.94	9.38 ± 2.32	24.47 ± 4.35	16.83
C16	Kaempferol-(galloylhexoside)-hexoside	N.D.	N.D.	11 ± 0.71	N.D.	2.75	N.D.	N.D.	N.D.	N.D.	N.D.	N.D.	N.D.	5.31 ± 0.95	1.77
C17	Kaempferol-hexoside-deoxyhexoside II	161.24 ± 4.9	76.88 ± 10.33	83.26 ± 1.96	142.54 ± 11.81	115.98	20.53 ± 8.33	26.62 ± 5.11	21.66 ± 4.63	23.14 ± 4.32	22.99	10.9 ± 4.31	7.3 ± 0.98	21.52 ± 4	13.24
C18	Kaempferol-rutinoside-hexoside	N.D.	N.D.	N.D.	N.D.	N.D.	N.D.	N.D.	N.D.	N.D.	N.D.	46.5 ± 7.37	21.92 ± 2.23	28.97 ± 7.1	32.46
C19	Kaempferol-hexoside	600.85 ± 9.2	296.91 ± 27.92	596.85 ± 15.93	541.8 ± 31.36	509.10	140.87 ± 50.59	165.57 ± 19.74	107.77 ± 11.66	143.01 ± 15.74	139.31	296.08 ± 92.14	152.75 ± 24.37	410.04 ± 43.89	286.29
C20	Kaempferol-hexoside II	502.67 ± 12.35	615.48 ± 29.92	648.55 ± 18.3	483.43 ± 22.92	562.53	124.52 ± 37.23	182.36 ± 24.96	253.4 ± 28.14	155.54 ± 22.47	178.95	252.15 ± 78.91	237.25 ± 36.35	360.41 ± 36.63	283.27
C21	Kaempferol or Luteolin-(galloylhexoside)-gallic a	7.98 ± 0.26	4.8 ± 0.77	11.61 ± 0.37	7.2 ± 0.31	7.90	2.09 ± 0.66	2.27 ± 0.17	1.78 ± 0.27	2.32 ± 0.2	2.11	2.68 ± 0.95	1.44 ± 0.2	4.73 ± 0.89	2.95
C22	Kaempferol-malonyl-hexoside I	256.97 ± 7	236.27 ± 18.67	363.32 ± 14.4	260.49 ± 22.57	279.26	248.94 ± 82.84	360.49 ± 42.62	387.15 ± 51.52	313.9 ± 39.74	327.62	60.17 ± 22.62	32.13 ± 7.21	87.52 ± 13.76	59.94
C23	Kaempferol-malonyl-hexoside II	256.97 ± 7	236.27 ± 18.67	363.32 ± 14.4	260.49 ± 22.57	279.26	248.94 ± 82.84	360.49 ± 42.62	387.15 ± 51.52	313.9 ± 39.74	327.62	60.17 ± 22.62	32.13 ± 7.21	87.52 ± 13.76	59.94
C24*	Kaempferol	25.25 ± 1.19	19.87 ± 2.78	78.98 ± 3.27	25.24 ± 2.58	37.33	2.8 ± 1.16	7.71 ± 1.76	11.98 ± 2.35	6.38 ± 1.32	7.22	20.59 ± 8.18	19.32 ± 4.12	33.83 ± 4.03	24.58
C25	Astragalin	502.67 ± 12.35	615.48 ± 29.92	648.55 ± 18.3	483.43 ± 22.92	562.53	124.52 ± 37.23	182.36 ± 24.96	253.4 ± 28.14	155.54 ± 22.47	178.95	252.15 ± 78.91	237.25 ± 36.35	360.41 ± 36.63	283.27
C26	Isorhamnetin-trihexoside	11.35 ± 0.24	11.1 ± 0.2	7.31 ± 5.66	11.15 ± 0.52	10.23	10.55 ± 5.18	11.51 ± 0.46	11.91 ± 0.77	11.68 ± 0.55	11.41	231.45 ± 42.07	231.85 ± 18.81	135.42 ± 20.72	199.57
C27	Isorhamnetin-trihexoside II	30.23 ± 1.67	11.43 ± 0.3	23.45 ± 1.06	25.81 ± 2.41	22.73	21.68 ± 4.23	16.16 ± 1.82	2.1 ± 5.15	16.22 ± 2.07	14.04	44.77 ± 6.98	35.64 ± 2.17	30.45 ± 4.81	36.95
C28	Isorhamnetin-(malonyl)-hexoside- hexoside	1003.63 ± 53.94	113 ± 10.51	502.95 ± 22.5	954.84 ± 110.39	643.61	617.3 ± 327.52	660.79 ± 157.32	413.32 ± 89.95	563.76 ± 119.77	563.79	54.76 ± 11.05	67.98 ± 2.75	53.55 ± 5.22	58.76
C29	Isorhamnetin-trihexoside III	16.35 ± 0.97	39.18 ± 1.38	60.27 ± 3.07	19.16 ± 1.73	33.74	33.58 ± 10.54	33.73 ± 4.1	58.6 ± 2.86	31.39 ± 4.22	39.33	141.37 ± 29.32	181.53 ± 5.96	126.15 ± 23.75	149.68
C30	Isorhamnetin-deoxyhexoside_-hexoside	1067 ± 47.7	195.38 ± 13.79	249.08 ± 11.12	920.44 ± 97.03	607.97	203.72 ± 60.13	232.51 ± 34.84	144.78 ± 29.79	195.83 ± 34.01	194.21	138.41 ± 8.73	482.66 ± 14.83	118.72 ± 8.89	246.60
C31	Isorhamnetin-(malonyl)-dihexoside	27.78 ± 1.01	12.41 ± 0.31	28.39 ± 1.92	24.83 ± 1.6	23.35	42.16 ± 18.7	40.96 ± 4.76	37.73 ± 4.33	38.13 ± 5.64	39.75	717.76 ± 117.99	385.8 ± 19.82	387.93 ± 82.8	497.16
C32	Isorhamnetin-deoxyhexoside-hexoside II	733.38 ± 499.45	137.91 ± 98.24	249.08 ± 11.12	459.81 ± 511.18	395.04	178.09 ± 96.99	113.9 ± 106.19	139.07 ± 27.19	140.09 ± 96.69	142.79	138.41 ± 8.73	482.67 ± 14.83	118.72 ± 8.89	246.60
C33	Isorhamnetin-digallic acid-benzoic acid	27.77 ± 1.02	12.41 ± 0.31	28.38 ± 1.92	24.82 ± 1.62	23.35	42.17 ± 18.72	40.97 ± 4.76	37.74 ± 4.33	38.13 ± 5.64	39.75	717.76 ± 117.99	385.8 ± 19.82	387.91 ± 82.78	497.15
C34	Isorhamnetin-deoxyhexoside_-hexoside III	79.95 ± 15.4	12.15 ± 1.53	84.83 ± 4.65	77.45 ± 13.83	63.60	21.88 ± 4.98	23.95 ± 2.69	27.14 ± 2.73	21.72 ± 2.42	23.67	55.28 ± 6.28	88.21 ± 7.94	36.34 ± 4.4	59.94
C35	Isorhamnetin-(p-coumaroyl)-hexoside	15.56 ± 1.89	12.39 ± 0.35	21.2 ± 3.41	13.31 ± 1.48	15.62	23.57 ± 7	26.23 ± 3.03	46.43 ± 8.08	23.63 ± 2.51	29.96	34.66 ± 4.45	76.22 ± 21.94	26.85 ± 2.53	45.91
C36	Quercetin-(galloyl)-dihexoside I	22.49 ± 0.89	12.03 ± 0.37	16.98 ± 0.39	21.11 ± 0.69	18.15	17.25 ± 2.04	20.63 ± 1.5	18.16 ± 2.18	19.28 ± 1.42	18.83	14.49 ± 0.18	19.03 ± 0.42	14.55 ± 0.44	16.03
C37	Quercetin-(galloyl)-dihexoside II	44.07 ± 1.13	12.61 ± 0.72	50.98 ± 2.39	41.88 ± 2.67	37.38	39.02 ± 10.82	47.92 ± 6.16	37.22 ± 3.79	43.69 ± 5.55	41.96	16.68 ± 0.75	25.07 ± 0.89	18.19 ± 1.04	19.98
C38	Quercetin-pentoside I	51.55 ± 25.52	44.22 ± 3.21	87.22 ± 4.82	50.28 ± 4.49	58.32	29.42 ± 8.53	34.42 ± 4.4	26.17 ± 2.01	31.17 ± 3.57	30.30	34.63 ± 8.75	32.73 ± 3.92	50.35 ± 5.98	39.24
C39	Quercetin-hexoside-deoxyhexoside	79.78 ± 6.29	71.01 ± 9.73	125.35 ± 18.3	64.21 ± 5.19	85.09	91.76 ± 39.69	106.48 ± 14.32	111.69 ± 20.39	95.7 ± 14.3	101.41	50.79 ± 11.09	42.74 ± 5.22	70.49 ± 8.02	54.67
C40	Quercetin-(galloyl)-hexoside	35.92 ± 2.82	23.94 ± 3.22	22.55 ± 6.07	31.13 ± 1.92	28.39	271.87 ± 78.13	185.93 ± 16.87	123.57 ± 15.8	179.81 ± 24.84	190.29	27.38 ± 6.17	17.79 ± 2.92	31.33 ± 5.18	25.50
C41	Quercetin-deoxyhexoside-hexoside II	33.42 ± 5.91	29.07 ± 5.59	44.89 ± 13.04	30.41 ± 3.21	34.45	41.5 ± 13.29	47.93 ± 6.78	36.43 ± 3.59	43.76 ± 5.59	42.41	70.96 ± 6.13	69.25 ± 1.71	53.56 ± 4.34	64.59
C42	Quercetin-(malonyl)-hexoside	11.84 ± 0.67	13.98 ± 0.74	16.12 ± 5.57	12.69 ± 0.3	13.66	236.81 ± 118.06	164.98 ± 21.53	209.57 ± 62.43	154.23 ± 31.16	191.40	14.6 ± 1.93	14.55 ± 1.55	15.33 ± 1.16	14.82
C43	Quercetin-(digalloyl)-hexoside	333.01 ± 17.6	44.45 ± 3.23	173.77 ± 6.67	294.46 ± 16.73	211.42	169.8 ± 69.94	163.69 ± 18.99	123.99 ± 13.92	159.73 ± 19.34	154.30	37.56 ± 8.44	36.41 ± 3.37	48.02 ± 3.22	40.66
C44	Quercetin-pentoside II	96.6 ± 7.5	38.33 ± 5.44	98.12 ± 7.41	83.29 ± 12.57	79.09	25.27 ± 5.81	26.4 ± 4.22	24.63 ± 3.9	25.26 ± 4.11	25.39	13.58 ± 0.78	15.48 ± 0.65	13.31 ± 0.48	14.12
C45*	Quercetin	154.67 ± 9.36	66.93 ± 4.23	153.63 ± 5.29	156.86 ± 11.89	133.02	47.58 ± 17.3	76.52 ± 9.72	95.16 ± 18.56	63.91 ± 9.63	70.79	20.73 ± 1.17	38.21 ± 1.91	21.2 ± 0.31	26.71
C46	Luteolin-(galloylhenxoside)-hexoside	484.53 ± 13.28	343.17 ± 30.57	665.13 ± 30.83	448.46 ± 41.59	485.32	64.2 ± 17.48	80.06 ± 13.72	68.22 ± 18.77	69.81 ± 10.03	70.57	305.2 ± 112.71	238.34 ± 34.3	541.59 ± 79.99	361.71
C47*	Luteoloside	86.72 ± 9.77	204.38 ± 46.92	55.57 ± 43.67	85.27 ± 15.5	107.98	173.26 ± 42.84	187.51 ± 42.05	151.65 ± 36.22	153.99 ± 29.23	166.60	47.39 ± 14.48	N.D.	50.8 ± 5.78	32.73
C48	Luteolin-deoxyhexoside I	454.69 ± 37.46	566.42 ± 49.41	258.46 ± 21.26	384.18 ± 50.82	415.94	53.81 ± 33.48	106.04 ± 26.34	49.27 ± 10.11	82.55 ± 23.02	72.92	64.97 ± 22.16	38.24 ± 5.92	108.69 ± 14.58	70.63
C49	Luteolin-deoxyhexoside II	173.28 ± 7.8	110.47 ± 27.26	425.55 ± 35.67	207.09 ± 26.63	229.10	64.19 ± 24.5	125.33 ± 23.21	93.02 ± 16.17	114.03 ± 21.19	99.14	64.88 ± 14.86	74.45 ± 3.55	99.94 ± 11.1	79.76
C50	Laricitrin-trihenxoside	N.D.	N.D.	130.62 ± 5.84	N.D.	32.65	N.D.	12.23 ± 5.56	129.61 ± 21.8	12.8 ± 4.85	38.66	254.62 ± 28.39	617.1 ± 25.87	242.47 ± 14.75	371.40
C51	Chrysoeriol-hexoside	1540.02 ± 156.79	67.46 ± 20.85	1747.28 ± 279.68	1507.3 ± 166.58	1215.51	1376.78 ± 445.56	1412.56 ± 427.65	477.03 ± 215.93	1030.09 ± 265.95	1074.11	271.72 ± 53.85	226.46 ± 35.78	251.95 ± 50.38	250.04
C52	Chrysoerio-hexoside	647.89 ± 33.68	375.73 ± 15.8	871.4 ± 108.62	362.98 ± 34.61	564.50	278.28 ± 67.45	N.D.	306.7 ± 33.06	167.36 ± 184.2	188.08	110.9 ± 6	220.23 ± 17.12	123.34 ± 6.82	151.49
C53	Syringetin-(malonyl)-hexoside	N.D.	N.D.	26.52 ± 4.89	N.D.	6.63	247.96 ± 117.64	320.23 ± 58.21	239.8 ± 55.15	273.73 ± 51.81	270.43	1480.79 ± 240.42	698.29 ± 54.15	893.85 ± 226.54	1024.31
C54	Hydroxy-dimethoxy-flavanone-O-hexoside	6.14 ± 1.28	58.2 ± 3.88	15.33 ± 2.43	14.6 ± 3.19	23.57	25.69 ± 15.18	27.96 ± 12.78	20.85 ± 10.52	22.1 ± 11	24.15	25.14 ± 10.48	19.23 ± 3.26	61.38 ± 9.89	35.25
C55	Naringenin	141.81 ± 6.21	64.53 ± 11.24	154.9 ± 8.82	101.82 ± 13.06	115.77	39.72 ± 3.3	96.87 ± 3.93	49.42 ± 12.06	84.11 ± 3.39	67.53	72.89 ± 18.62	60.81 ± 5.84	116.34 ± 15.36	83.35
C56*	Taxifolin	11.35 ± 2.42	24.57 ± 3.52	21.6 ± 2.59	16.73 ± 2.46	18.56	35 ± 5.74	44.67 ± 6.35	44.81 ± 5.75	37.14 ± 5.26	40.40	13.36 ± 4.76	13.43 ± 4.74	7.46 ± 2.84	11.42
C57	Apigenin hexoside	33.33 ± 1.7	34.02 ± 4.22	27.39 ± 3.06	31.92 ± 3.33	31.67	17.63 ± 3.4	21.87 ± 2.83	12.61 ± 2.08	17.35 ± 1.82	17.36	83.05 ± 6.97	43.35 ± 10.26	75.88 ± 5.06	67.43
C58*	Apigenin	7.86 ± 1.55	4.69 ± 0.93	11.07 ± 2.16	6.83 ± 1.23	7.61	4.57 ± 2.46	8.12 ± 2.43	8.51 ± 2.01	6.75 ± 2	6.99	7.67 ± 1.25	5.34 ± 1.51	8.37 ± 0.68	7.13
C59	Aucubin	N.D.	70.12 ± 6.09	12.34 ± 1.47	N.D.	20.62	N.D.	26.3 ± 3.54	13.34 ± 1.97	20.36 ± 3.44	15.00	35.07 ± 8.49	66.26 ± 4.56	22.36 ± 5.92	41.23
C60	Mudanpioside E	N.D.	34.51 ± 5.54	23.42 ± 5.55	N.D.	14.48	14.51 ± 10.74	9.62 ± 8.01	23.56 ± 11.96	8.14 ± 6.61	13.96	50.92 ± 3.61	277.21 ± 20.59	50.13 ± 3.84	126.09
C61	Isomaltopaeoniflorin	74.81 ± 5.13	8.13 ± 0.62	43.48 ± 2.9	69.22 ± 3.82	48.91	29.59 ± 15.7	29.77 ± 5.75	33.33 ± 7.75	27.4 ± 5.89	30.02	20.98 ± 1.42	32.66 ± 0.97	21.36 ± 1.68	25.00
C62	Oxypaeoniflora III	275.9 ± 17.7	37.52 ± 2.47	112.89 ± 27.89	251.77 ± 23.69	169.52	20.48 ± 4.17	17.76 ± 1.6	13.12 ± 3.73	18.14 ± 2.89	17.37	504.18 ± 89.61	199.38 ± 79.91	407.55 ± 15.51	370.37
C63	Oxypaeoniflora IV	362.77 ± 26.5	73.95 ± 10.92	120.3 ± 6.61	315.43 ± 19.41	218.11	65.38 ± 8.87	45.25 ± 6.34	51.54 ± 16.32	44.81 ± 6.82	51.75	664.3 ± 79.49	371.63 ± 46.59	565.9 ± 21.7	533.94
C64*	Galloylpaeoniflorin I	28.58 ± 1.91	35.06 ± 2.41	64.81 ± 5.67	37.05 ± 1.75	41.38	45.61 ± 12.58	101.89 ± 19.66	133.32 ± 31.02	113.69 ± 19.06	98.63	40.67 ± 3.4	438.37 ± 43.43	53.39 ± 4.67	177.48
C65	Mudanpioside H	11.27 ± 6.43	N.D.	6.66 ± 0.66	7.18 ± 1.15	6.28	N.D.	N.D.	7.2 ± 2.25	2.94 ± 1.23	2.54	N.D.	11.76 ± 1.22	N.D.	3.92
C66	Galloylpaeoniflorin II	45.49 ± 1.52	N.D.	25.69 ± 1.61	39.93 ± 3.05	27.78	18.63 ± 4.93	20.9 ± 2.01	9.98 ± 2.08	25.27 ± 2.41	18.70	10.82 ± 2.04	25.69 ± 5.28	15.8 ± 2.5	17.44
C67	Benzoyloxypaeoniflflorin	8.38 ± 1.76	33.1 ± 12.08	34.67 ± 2.26	5.84 ± 1.38	20.50	N.D.	N.D.	N.D.	0.06 ± 0.16	0.02	49.26 ± 14.09	62.52 ± 7.91	31.08 ± 3.91	47.62
C68	Mudanpioside J	46.08 ± 2.39	185.35 ± 4.9	34.69 ± 6.09	49.86 ± 4.97	78.99	93.98 ± 31.07	93.99 ± 27.95	78.42 ± 29.61	81.66 ± 24.48	87.01	189.15 ± 28.94	68.04 ± 15.36	189.94 ± 37.83	149.04
C69	Mudanpioside B	46.08 ± 2.39	185.35 ± 4.9	34.69 ± 6.09	46.07 ± 3.78	78.05	70.81 ± 20.58	78.5 ± 12.52	43.75 ± 39.32	67.4 ± 8.61	65.11	189.15 ± 28.94	21.09 ± 30.29	154.93 ± 9.67	121.73

*Targets were quantified by using reference compounds.

Multivariate statistical analysis based on the quantification data was further carried out. As shown in **Figure 4B**, the PCA score plot shows three distinct clusters (R^2^X = 0.901 and Q^2^ = 0.830). The detailed content of each target among the 66 samples was for hierarchical cluster analysis using the Ward chain algorithm. In the heat map and dendrogram (**Figure 4C**), it graphically showed that all the samples could be divided into three major clusters (stamen, calyx, and petal). each major cluster can be roughly separated into cultivars sub-clusters (SD, AH, SX and SC). Given the fact that there is conceptual disparity between targeted and untargeted metabolomics analysis, our data showed very consistency between these two methods. Within the petal group, SC samples were clustered separately, while AH, SD, and SX samples were clustered together, which is perfectly fit into the color discrepancies of the four cultivar petals (**Figure 1**). This finding suggested a clear correlation between those 69 major components and petal color.

As one of major natural colorants, anthocyanins play important role in color formation during plant blooming. Our subsequent quantitative analysis of individual components showed that the total content of 12 major anthocyanins individuals was in line with the total anthocyanins content (TAC). Among all petals of the four cultivars, the SC samples contained fewer anthocyanins than the other cultivars. For individual anthocyanins, the contents were lower in SC petals than other cultivars. For instance, the content of Cy-di-glu, one of the main anthocyanins in petals, were nearly 100 times lower in SC petals than others. However, the content of Pt-glu was relative higher in SC petals (2.53 ± 0.28 μg/mL) than AH (0.36 ± 0.02 μg/mL) and SX (0.51 ± 0.05 μg/mL). Wang et al’s research (Yue et al., 2023) suggested that Pg-glu was responsible for red color in CPF petal. From our data, however, the contents of Pg-glu (0~0.084 μg/mL) were too low to be detected in most of samples. The major anthocyanins among four cultivar petal samples are Cy-di-glu, Cy, Pn-glu and Cy- malonyl glu, of which the contents are significant lower in SC petals than others. It’s suggested that Cy and its glucosides mainly contributed to the color discrepancy. In addition, our quantification data suggested that some of the major flavonoid glycoside may contribute to the lighter color of SC petals as well, as those flavonoid compounds were significant lower that other three cultivar petals (**Supplementary Table S2**).

We also quantified the relative contents of ten monoterpenoids. It was found that the content of mudanpioside in the petals of the SC cultivar was much higher than that of other cultivars, while other terpenoids contents were the opposite. In addition, the content of galloylpaeoniflorin in SD cultivars was overall higher than those cultivars, especially in the stamen part.

### 3.4 Antioxidant activities and total phenolic, flavonoid, and anthocyanin contents

**Figure 5:**
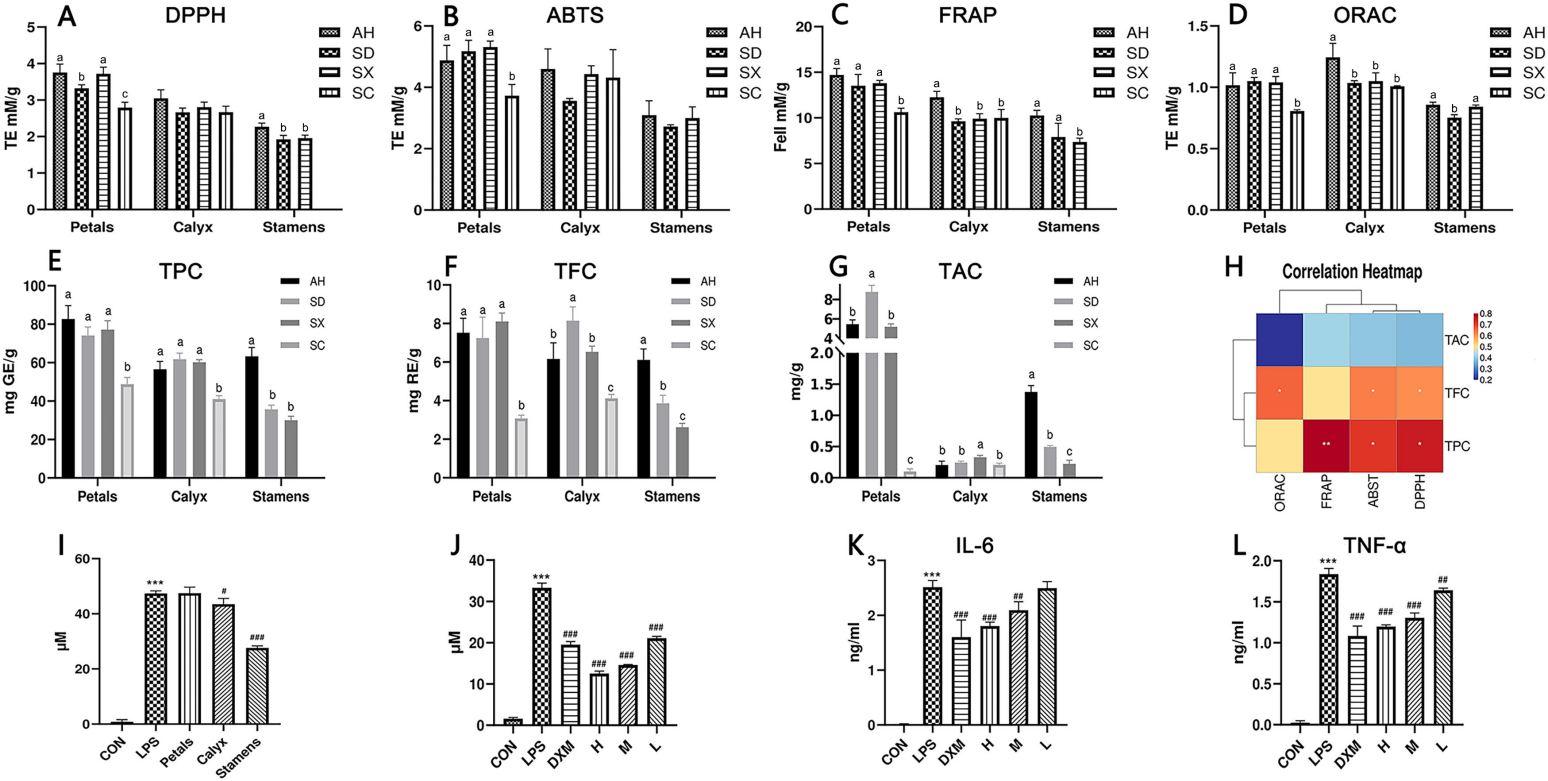
**(A–D)** Antioxidant capacity results, data are presented as mean ± SD (n = 3). Different superscript letters in each row indicate significant differences. Using one-way ANOVA or Tamhane T^2^, ^abc^ *p*<0.05. **(E–G)** Total polyphenol, total flavonoid, and total anthocyanin contents of different species and parts (n = 3), Using one-way ANOVA, ^abc^ *p*<0.05. **(H)** Correlation heat map between total polyphenols, total flavonoids, total anthocyanins content, and antioxidant capacity. correlation coefficients present as the color. **(I)** Effects of the petals, calyx, and stamens of CPF on inflammatory extracellular NO release (n = 3). Effects of CPF stamen extracts at 93.75, 46.88, and 23.44 μg/mL concentrations on the release of extracellular NO **(J)**, secretion of IL-6 **(K)** and TNF-α **(L)** (n = 3). ^#^, ^##^, and ^###^ denote *p*< 0.05, 0.01, and 0.001, respectively compared with the LPS group. ***p< 0.001 compared with control group.

Reactive oxygen species (ROS) are widely present in cells. Excess ROS usually causes oxidative stress, leading to various diseases such as inflammation, cancer, and aging. The antioxidant activity of different parts of CPF is measured by ABTS, DPPH, FRAP, and ORAC methods, respectively. Among them, ABTS, DPPH, and FRAP are used to obtain antioxidant capacity through the single electron transfer (SET) reaction mechanism, while the ORAC method is used to obtain antioxidant capacity through the hydrogen atom transfer (HAT) reaction mechanism. HAT is the key step in the free radical chain reaction. Therefore, those reacting with hydrogen atom transfer are more effective in terms of free radical-breaking antioxidant capacity (Huang et al., 2005; Brewer, 2011). In general, all the samples showed good antioxidant activities. Although the data obtained differed, the three SET antioxidant assays (ABTS, DPPH, and FRAP) showed a parallel trend (**Figures 5A–D**). For SET mechanism antioxidant assays, petals samples showed relative stronger activity than other two parts. Among petal samples, SC cultivars showed the weakest antioxidant activities, regardless of the method applied, while other three cultivars were at same levels. It indicated a significant correlation between the petal colors and the antioxidant activities.

As shown in **Figures 5E–G**, the TPC content ranged from 30.01 to 82.74 mg (RE)/g. The TFC contents in CPF petals, calyx, and stamens ranged from 3.08 - 8.10 mg (RE)/g, 4.11 - 8.14 mg (RE)/g, and 0.22 - 1.37 mg (RE)/g, respectively. TAC ranges from 0.10 mg/g DW to 8.78 mg/g DW. The TAC content results indicated that most anthocyanins exist in petals other than in calyx and stamens. Overall, the TPC, TAC, and TFC of SC samples were significantly lower than other cultivars, which strongly correlated with their shade of color. The different distribution patterns of TPC, TFC, and TAC in CPF may be due to interspecific biodiversity.

The correlation between TPC, TAC, and TFC and antioxidant capacity in CPF was also analyzed (**Figure 5H**). It showed that the contents of anthocyanins, phenolics and flavonoids had a positive correlation with three all the antioxidant capacity tests (FRAP, ABST, ORAC), however there is no significant relation between the contents and the DPPH test. In general, our correlation analysis suggests that the TAC, TPC and TFC had positive but not negative correlation with the antioxidant activity.

### 3.5 Anti-inflammatory activity

The anti-inflammatory activity of the petals, calyx, and stamens of Anhui CPF was assessed using the LPS-induced RAW264.7 inflammation model. As shown in **Figure 5I**, only the stamens samples significantly reduced NO release from macrophages in a dose-dependent manner (**Figure 5J**). Further ELISA tests showed that it had a significant dose-dependent inhibitive effect on LPS-induced release of inflammatory factors IL-6 and TNF-α (**Figures 5K, L**). To our knowledge, this the first report of the anti-inflammation activity of CPF.

## 4 Discussion

Anthocyanins are major components in CPF petals. The correlation analysis among chemical quantification, petal color and antioxidant assays suggested a notion that the deeper the color is, the better antioxidant activity. For instance, the anthocyanins contents in SC cultivars (light pink and white) were much lower than other deep-color cultivars (deep purple or reddish purple), Correspondingly, their antioxidant activities (**Figure 5**) were also the lowest in all the four petals. For individual anthocyanins, cyanidin (cy)-type (C1, C4, C5, C7 and C12) and peonidin (pn)-type anthocyanins (C10) had positive relation with antioxidant activities, while pelargonidin (C6), petunidin (C8) and malvidin (C11) types has negative impact on antioxidant activities.

The stamens samples consist of lower phenolics, flavonoids and anthocyanins contents. However, our quantification data showed that their contents of monoterpenoid such as oxypaeoniflora, galloylpaeoniflorin, mudanpioside J and mudanpioside B were significantly higher than other parts. This clearly indicates that its superior anti-inflammatory effects are more attributed to these monoterpenoid components other than polyphenolic compounds.

In this paper, we explored the potential applications of Chinese peony flowers, particularly focusing on their chemical and biological properties. According to our experiment design, environment factors were managed to avoid by using vigorous sample collection protocol, by which sample were harvested at same experimental field and same time. It dramatically increased the sampling homogeneity, reduced in-sample chemical variations, and hence facilitated our understanding about the inherent chemical variation among those commonly grown cultivars in China. It, however, may not accurately represent the variability of peony species and their uses in different climates and cultures.

In summary, our comprehensive chemical and biological investigation indicated that CPF is rich in bioactive phytochemicals, and possess potential health benefits, including anti-inflammatory, and antioxidant effects. Previous *in vivo* studies also indicated *Paeonia* petals could alleviate oxidative stress and restore gut microbiota (Liu et al., 2021), and exert skin-beneficial effects (Cutovic et al., 2022) by antioxidant, antimicrobial and antibiofilm activities. Combining those findings, it supports the potential use of CPF as a functional bioactive ingredient. Moreover, the revalorization of CPF could be diversified with different application purpose.

## Data Availability

The original contributions presented in the study are included in the article/**Supplementary Material**. Further inquiries can be directed to the corresponding author.

## References

[B1] An H. M. Ou X. C. Zhang Y. B. Li S. Xiong Y. F. Li Q.. (2022). Study on the key volatile compounds and aroma quality of jasmine tea with different scenting technology (vol 385, 132718, 2022). Food Chem. 385, 132718. doi: 10.1016/j.foodchem.2022.133172 35313197

[B2] Bai Z. Z. Tang J. M. Ni J. Zheng T. T. Zhou Y. Sun D. Y.. (2021). Comprehensive metabolite profile of multi-bioactive extract from tree peony (Paeonia ostii and Paeonia rockii) fruits based on MS/MS molecular networking. Food Res. Int. 148, 110609. doi: 10.1016/j.foodres.2021.110609 34507753

[B3] Brewer M. S. (2011). Natural antioxidants: sources, compounds, mechanisms of action, and potential applications. Compr. Rev. Food Sci. Food Saf. 10, 221–247. doi: 10.1111/j.1541-4337.2011.00156.x

[B4] Chen J. Liu F. Wu R. A. Chen J. Wang W. Ye X.. (2022). An up-to-date review: differential biosynthesis mechanisms and enrichment methods for health-promoting anthocyanins of citrus fruits during processing and storage. Crit. Rev. Food Sci. Nutr. 64, 3989–4015. doi: 10.1080/10408398.2022.2137778 36322523

[B5] Cutovic N. Markovic T. Kostic M. Gasic U. Prijic Z. Ren X.. (2022). Chemical profile and skin-beneficial activities of the petal extracts of Paeonia tenuifolia L. from Serbia. Pharm. (Basel) 15, 1537. doi: 10.3390/ph15121537 PMC9787298 36558988

[B6] Duan X.-J. Zhang W.-W. Li X.-M. Wang B.-G. (2006). Evaluation of antioxidant property of extract and fractions obtained from a red alga, Polysiphonia urceolata. Food Chem. 95, 37–43. doi: 10.1016/j.foodchem.2004.12.015

[B7] Han X. Hu S. Yang Q. Sang X. Tang D. Cao G. (2022). Paeoniflorin ameliorates airway inflammation and immune response in ovalbumin induced asthmatic mice: From oxidative stress to autophagy. Phytomedicine 96, 153835. doi: 10.1016/j.phymed.2021.153835 34799185

[B8] Huang D. Ou B. Prior R. L. (2005). The chemistry behind antioxidant capacity assays. J. Agric. Food Chem. 53, 1841–1856. doi: 10.1021/jf030723c 15769103

[B9] Kang L. Miao J. X. Cao L. H. Miao Y. Y. Miao M. S. Liu H. J.. (2020). Total glucosides of herbaceous peony (Paeonia lactiflora Pall.) flower attenuate adenine- and ethambutol-induced hyperuricaemia in rats. J. Ethnopharmacol. 261, 113054. doi: 10.1016/j.jep.2020.113054 32534113

[B10] Kwak C. S. Lee M. S. Park S. C. (2007). Higher antioxidant properties of Chungkookjang, a fermented soybean paste, may be due to increased aglycone and malonylglyco side isoflavone during fermentation. Nutr. Res. 27, 719–727. doi: 10.1016/j.nutres.2007.09.004

[B11] Li P. Shen J. Wang Z. Liu S. Liu Q. Li Y.. (2021). Genus Paeonia: A comprehensive review on traditional uses, phytochemistry, pharmacological activities, clinical application, and toxicology. J. Ethnopharmacol. 269, 113708. doi: 10.1016/j.jep.2020.113708 33346027

[B12] Li Z. Ma Y. Li F. Wei Y. Zhang L. Yu L.. (2023). Quality evaluation of peony petals based on the chromatographic fingerprints and simultaneous determination of sixteen bioactive constituents using UPLC-DAD-MS/MS. Molecules 28, 7741. doi: 10.3390/molecules28237741 38067470 PMC10708337

[B13] Liu X. Chen Y. Zhang J. He Y. Ya H. Gao K.. (2022). Widely targeted metabolomics reveals stamen petaloid tissue of Paeonia lactiflora Pall. being a potential pharmacological resource. PloS One 17, e0274013. doi: 10.1371/journal.pone.0274013 36054136 PMC9439255

[B14] Liu L. Yuan Y. Tao J. (2021). Flavonoid-Rich Extract of Paeonia lactiflora Petals Alleviate d-Galactose-Induced Oxidative Stress and Restore Gut Microbiota in ICR Mice. Antioxidants 10, 1889. doi: 10.3390/antiox10121889 34942992 PMC8698645

[B15] Liu L. Yuan Y. Zuo J. Tao J. (2023). Composition and antioxidant activity of Paeonia lactiflora petal flavonoid extract and underlying mechanisms of the protective effect on H2O2-induced oxidative damage in BRL3A cells. Hortic. Plant J. 9, 335–344. doi: 10.1016/j.hpj.2022.06.001

[B16] Moldovan B. Hosu A. David L. Cimpoiu C. (2016). Total phenolics, total anthocyanins, antioxidant and pro-oxidant activity of some red fruits teas. Acta Chim. Slov. 63, 213–219. doi: 10.17344/acsi.2015.1421 27333542

[B17] Nie R. Zhang Y. Jin Q. Zhang S. Wu G. Chen L.. (2021). Identification and characterisation of bioactive compounds from the seed kernels and hulls of Paeonia lactiflora Pall by UPLC-QTOF-MS. Food Res. Int. 139, 109916. doi: 10.1016/j.foodres.2020.109916 33509483

[B18] Ogawa K. Nakamura S. Sugimoto S. Tsukioka J. Hinomaru F. Nakashima S.. (2015). Constituents of flowers of Paeoniaceae plants, Paeonia suffruticosa and Paeonia lactiflora. Phytochem. Lett. 12, 98–104. doi: 10.1016/j.phytol.2015.03.002

[B19] Qiu J. Chen X. Liang P. Zhang L. Xu Y. Gong M.. (2022). Integrating approach to discover novel bergenin derivatives and phenolics with antioxidant and anti-inflammatory activities from bio-active fraction of Syzygium brachythyrsum. Arabian J. Chem. 15, 103507. doi: 10.1016/j.arabjc.2021.103507

[B20] Qiu X. Zhang J. Huang Z. Zhu D. Xu W. (2013). Profiling of phenolic constituents in Polygonum multiflorum Thunb. by combination of ultra-high-pressure liquid chromatography with linear ion trap-Orbitrap mass spectrometry. J. Chromatogr A 1292, 121–131. doi: 10.1016/j.chroma.2012.11.051 23246090

[B21] Sang X. Wan X. Zhang H. Ying J. Wang L. Yang Q.. (2023). The most bioactive fraction of stir-fried Radix Paeoniae Alba regulating IL-6/STAT3 signaling pathway in allergic asthma mouse. J. Ethnopharmacol. 301, 115821. doi: 10.1016/j.jep.2022.115821 36220510

[B22] Shu X. K. Duan W. J. Liu F. Shi X. G. Geng Y. L. Wang X.. (2014). Preparative separation of polyphenols from the flowers of Paeonia lactiflora Pall. by high-speed counter-current chromatography. J. Chromatogr. B Anal. Technol. Biomed. Life Sci. 947, 62–67. doi: 10.1016/j.jchromb.2013.12.004 24406305

[B23] Wu F. Wu G. Li T. Lu W. Fu T. Zhang Z. (2023). Exploring the target and mechanism of radix Paeoniae alba on Sjogren’s syndrome. Comb. Chem. High Throughput Screen 26, 1224–1232. doi: 10.2174/1386207325666220823144054 36017844 PMC10236566

[B24] Xu Y. Liang P. L. Chen X. L. Gong M. J. Zhang L. Qiu X. H.. (2021). The impact of citrus-tea cofermentation process on chemical composition and contents of Pu-Erh tea: an integrated metabolomics study. Front. Nutr. 8. doi: 10.3389/fnut.2021.737539 PMC8484324 34604284

[B25] Xu J. J. Xu F. Wang W. Wang P. P. Xian J. Han X.. (2022). Paeoniae Radix Rubra can enhance fatty acid beta-oxidation and alleviate gut microbiota disorder in alpha-naphthyl isothiocyanate induced cholestatic model rats. Front. Pharmacol. 13. doi: 10.3389/fphar.2022.1002922 PMC9633937 36339580

[B26] Yang S. Du Q. Q. Yue Q. X. Sun Y. F. Jin C. S. Zhang W.. (2023). Analysis and evaluation on Paeoniae Radix Alba from different cultivars by UPLC-Q-TOF-MS and HPLC. Zhongguo Zhong Yao Za Zhi 48, 715–724. doi: 10.19540/j.cnki.cjcmm.20220618.201 36872235

[B27] Yuan Q. L. (2011). The culture of Chinese herbaceouspeony (Beijing: Beijing Forestry University).

[B28] Yue F. Zhang F. Qu Q. Wang C. Qin Y. Ma L.. (2023). Effects of ageing time on the properties of polysaccharide in tangerine peel and its bacterial community. Food Chem. 417, 135812. doi: 10.1016/j.foodchem.2023.135812 36921363

[B29] Yun-Jie L. I. Hua-Jun F. U. Bo Y. U. Chen W. Wang Y. K. (2019). Nutritional function analysis of Chinese scented tea and research progress on its main processing technology. Storage Process. 19, 207–210.

[B30] Zhang J. Wu X. Qiu J. Zhang L. Zhang Y. Qiu X.. (2020a). Comprehensive comparison on the chemical profile of Guang Chen Pi at different ripeness stages using untargeted and pseudotargeted metabolomics. J. Agric. Food Chem. 68, 8483–8495. doi: 10.1021/acs.jafc.0c02904 32610017

[B31] Zhang J. Xu Y. Ho C. T. Qiu J. Q. Qiu X. H. Huang Z. H.. (2022). Phytochemical profile of Tibetan native fruit “Medog lemon” and its comparison with other cultivated species in China. Food Chem. 372, 131255. doi: 10.1016/j.foodchem.2021.131255 34627084

[B32] Zhang Y. Zhang Y. Duan X. Liu X. Yuan S. Han J.. (2020b). Anthocyanins in tree peony (Paeonia suffruticosa) and their relationship with flower color. Hortic. Sci. Technol. 38, 776–784. doi: 10.7235/hort.20200070

[B33] Zhao Q. Gu L. Li Y. Q. Zhi H. Luo J. R. Zhang Y. L. (2023). Volatile composition and classification of Paeonia lactiflora flower aroma types and identification of the fragrance-related genes. Int. J. Mol. Sci. 24, 9410. doi: 10.3390/ijms24119410 37298360 PMC10253308

