# Supplementary material for: Comprehensive chemical and bioactive investigation of Chinese peony flower: a case of valorization of by-products as a new food ingredient from Chinese herb
Source: Front Plant Sci. 2025 Jan 27;15:1501966. doi: 10.3389/fpls.2024.1501966 (PMC11808149; doi:10.3389/fpls.2024.1501966)

Figure S1 The proposed MS fragmentations of methyl-tetra-galloyl hexoside, Isomaltopaeoniflorin, and isorhamnetin-(p-coumaroyl)-hexoside.


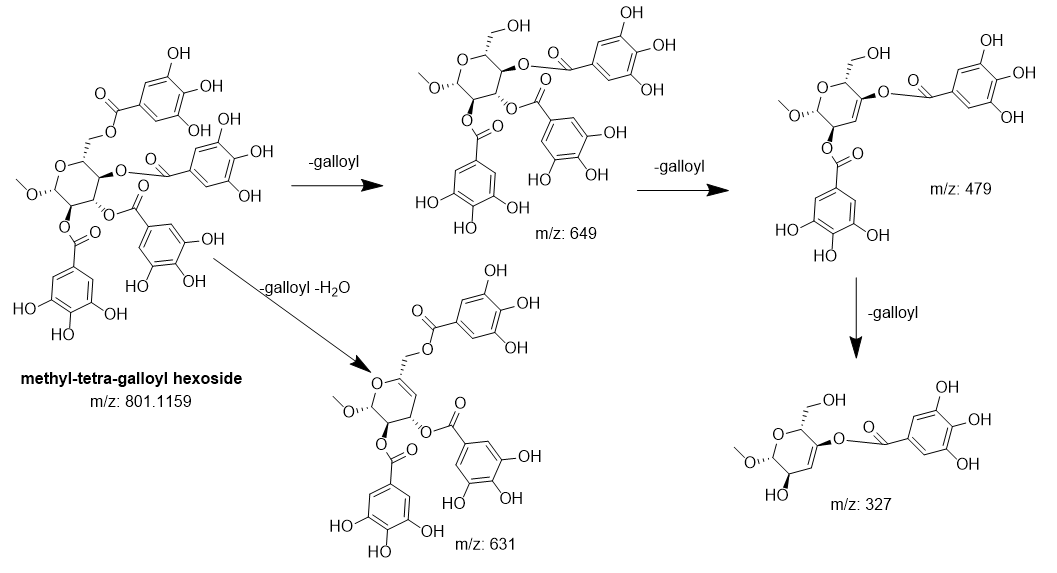


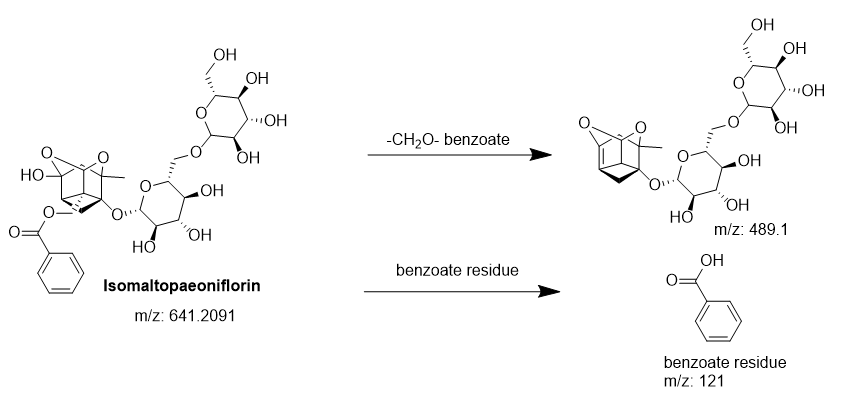


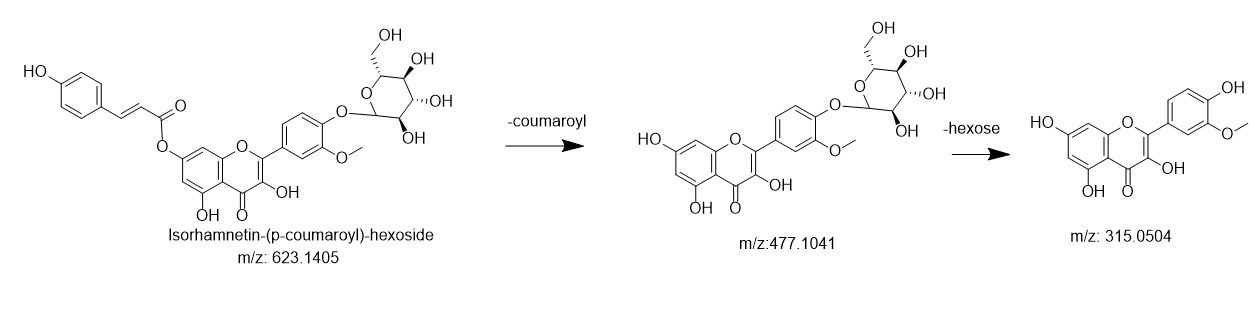

Supplement: Supplementary file 1 [file SupplementaryFile1.docx]
