# Supplementary material for: Comprehensive chemical and bioactive investigation of Chinese peony flower: a case of valorization of by-products as a new food ingredient from Chinese herb
Source: Front Plant Sci. 2025 Jan 27;15:1501966. doi: 10.3389/fpls.2024.1501966 (PMC11808149; doi:10.3389/fpls.2024.1501966)
Supplement: Supplementary file 2 [file Table1.docx]

***Supplementary Material***

**Supplementary Tables**

Table S1，Methodological validation results

| **Standard** | **Linear equation** | **Linear range**  **（ng/ml）** | **R^2^** | **LOQ**  **（ng/ml）** | **LOD**  **（ng/ml）** | **Precision (RSD, %)** | | | **Recovery** | |
| --- | --- | --- | --- | --- | --- | --- | --- | --- | --- | --- |
|  |  |  |  |  |  | **Intraday** | **Interday** | **Intermediate precision** | **Recovery rate (%)** | **RSD (%)** |
| Cy-3-O-glu | Y=398824X-1358763 | 4000-15.63 | 0.9994 | 1.0 | 0.4 | 1.43,1.14,1.98 | 1.80,4.32,2.31 | 5.35 | 85.71±1.27 | 1.48 |
| Pn-3-O-glu | Y=134598X-3406732 | 8000-31.25 | 0.9995 | 0.6 | 0.2 | 1.61,3.20,2.44 | 2.02,4.24,2.63 | 4.46 | 100.64±4.94 | 4.91 |
| Mv-3-O-glu | Y=186252X-2124181 | 1000-15.625 | 0.9979 | 1.0 | 0.6 | 2.26,2.53,2.32 | 1.69,4.91,2.77 | 3.81 | 98.33±5.04 | 5.13 |
| Pt-3-O-glu | Y=66415X-1498781 | 1000-3.90 | 0.9980 | 2.0 | 0.6 | 2.50,2.26,4.61 | 5.01,6.55,3.92 | 1.56 | 93.61±5.59 | 5.89 |
| Pg-3-O-glu | Y=379806X-2419677 | 1000-3.90 | 0.9997 | 1.0 | 0.4 | 2.99,3.62,3.44 | 3.23,5.12,4.54 | 8.56 | 95.74±8.54 | 7.01 |
| Dp-3-O-glu | Y=72822X-1968012 | 5000-19.53 | 0.9991 | 0.6 | 0.6 | 1.36,0.30,2.13 | 3.97,2.49,3.20, | 6.12 | 99.38±0.70 | 0.70 |
| Cy-3-O-ruti | Y=177241X-427241 | 500-1.95 | 0.9999 | 0.4 | 0.4 | 3.54,4.06,3.13 | 3.40,5.35,3.42 | 3.55 | 99.45±9.54 | 9.60 |
| Cy-3-O-(6''-malglu | Y=31435X-775968 | 1500-11.72 | 0.9953 | 1.0 | 0.6 | 3.73,2.67,3.70 | 6.29,5.33,6.47 | 11.25 | 112.09±10.99 | 9.81 |
| Quercetin | Y=1088216X-11446266 | 1500-5.86 | 0.9961 | 1.0 | 0.6 | 0.67,4.79,2.86 | 2.78,5.30,6.54 | 3.56 | 108.92±3.87 | 3.55 |
| Apigenin | Y=539876X+560944 | 500-1.95 | 0.9998 | 1.0 | 0.4 | 3.51,2.39,3.99 | 3.00,7.56,3.79, | 6.55 | 92.64±2.63 | 2.84 |
| Luteoloside | Y=245616X+1031527 | 2000-7.81 | 0.9973 | 2.0 | 0.6 | 1.72,2.47,1.64 | 1.84,3.29,1.93 | 7.24 | 96.53±10.26 | 10.62 |
| Kaempferol | Y=463575X+23487480 | 8000-31.25 | 0.9987 | 0.8 | 0.2 | 1.83,2.86,2.50 | 2.17,1.11,1.42 | 4.01 | 93.41±3.44 | 3.69 |
| Taxifolin | Y= 390875X + 705271 | 128-2 | 0.9975 | 0.8 | 0.2 | 4.48,3.84,4.02 | 4.13,5.66,4.36 | 5.99 | 100.68±3.52 | 3.50 |
| 6'-O-Galloyl paeoniflorin | Y= 4203546X+ 22015213 | 560-2.19 | 0.9980 | 1.0 | 0.1 | 0.98,0.31,0.81 | 4.66,6.28,4.29 | 9.05 | 94.17±3.80 | 4.03 |
