# Supplementary material for: Comprehensive chemical and bioactive investigation of Chinese peony flower: a case of valorization of by-products as a new food ingredient from Chinese herb
Source: Front Plant Sci. 2025 Jan 27;15:1501966. doi: 10.3389/fpls.2024.1501966 (PMC11808149; doi:10.3389/fpls.2024.1501966)
Supplement: Supplementary file 4 [file Table3.docx]

**Table S3.** Components identified or tentatively characterized in the flower of Chinese Peony.

| **No.** | **RT** | **[M-H]^–^**  **(ppm)** | **[M+H]^+^**  **(ppm)** | **Molecular formula** | **Characteristic Fragment ions ^-^** | **Characteristic Fragment ions ^+^** | **Identification** |
| --- | --- | --- | --- | --- | --- | --- | --- |
| **Gallic acid derivatives** | | | | | | | |
|  | 1.85 | 331.0671  (0.031) | 333.0811  (-1.420) | C_13_H_16_O_10_ | 271.0459, **169.0131**, 151.0025,**125.0230** | **171.0289**, 153.0186 | galloyl hexoside(Cutovic et al., 2022) |
|  | 2.36 | 169.0131  (-6.725) | 171.0287  (-0.584) | C_7_H_6_O_5_ | **125.0230**, 97.0281 | **127.0390**, 81.0450 | gallic acid |
|  | 2.67 | 345.0823  (-1.188) | 347.0963  (-2.689) | C_14_H_18_O_10_ | 285.0622, 225.0396, **183.0288** | 227.5673, **185.0999** | methyl gallate-hexoside I(Cutovic et al., 2022) |
|  | 2.90 | 483.0782  (0.335) | 485.0911  (-2.992) | C_20_H_20_O_14_ | **331.0676**, 313.0559, **169.0130** | N.D. | di-galloyl hexoside |
|  | 2.93 | 325.0562  (0.687) | 327.0712  (0.687) | C_14_H_14_O_9_ | 173.0445, **169.0131** | N.D. | galloyl shikimic acid |
|  | 2.98 | 345.0822  (-1.565) | 347.0967  (-1.709) | C_14_H_18_O_10_ | 285.0613, 225.0402, **183.0288** | 227.5673, **185.0999** | methyl gallate-hexoside II(Marcelino et al., 2023) |
|  | 3.26 | 783.0682  (-0.518) | N.D. | C_34_H_24_O_22_ | **481.0613, 300.9988, 275.0199, 249.0400,** | N.D. | di-HHDP-hexoside(Milczarek et al., 2021) |
|  | 3.38 | 315.0720  (-0.524) | 317.0871  (1.329) | C_13_H_16_O_9_ | **169.0131**, 151.0023 | N.D. | galloyl deoxyhexoside |
|  | 3.71 | 483.0782  (0.335) | 485.0935  (1.976) | C_20_H_20_O_14_ | **331.0666**, **169.0131** | N.D. | di-galloyl hexoside II |
|  | 4.96 | 783.0682  (-0.518) | N.D. | C_34_H_24_O_22_ | **481.0613, 300.9988, 275.0199, 249.0400,** | N.D. | di-HHDP-hexoside(Milczarek et al., 2021) |
|  | 5.39 | 483.0783  (0.583) | 485.0924  (-0.292) | C_20_H_20_O_14_ | **331.0663**, 313.0562, 271.0462, 211.0241, **169.0131**, 125.0227 | **171.0280**,153.0184 | di-galloyl hexoside Ⅲ(Marcelino et al., 2023) |
|  | 5.61 | 321.0250  (-0.670) | 323.0393  (-1.481) | C_14_H_10_O_9_ | **169.0131, 125.0229** | 153.0182 | galloyl gallic acid(Cutovic et al., 2022) |
|  | 5.90 | 635.0891  (0.099) | N.D. | C_27_H_24_O_18_ | **465.0673**, **313.0565**, 169.0131, 125.0231 | N.D. | tri-galloyl hexoside(Cutovic et al., 2022) |
|  | 5.98 | 483.0782  (0.397) | 485.0927  (0.203) | C_20_H_20_O_14_ | **331.0663**, 313.0562, 271.0462, **169.0131**, 125.0227 | **171.0285**, 153.0181 | di-galloyl hexoside IV |
|  | 6.02 | 633.0737  (0.526) | N.D. | C_27_H_22_O_18_ | 463.0536, **300.9988, 275.0199, 249.0400,** | N.D. | galloyl-HHDP- hexoside |
|  | 6.11 | 321.0252  (-0.172) | 323.0392  (-1.883) | C_14_H_10_O_9_ | **169.0131, 125.0230** | 153.0182 | galloyl gallic acid II(Marcelino et al., 2023) |
|  | 6.18 | 483.0782  (0.397) | 485.0931  (1.089) | C_20_H_20_O_14_ | 439.0882,**331.0668**, 313.0564, **169.0131**, 125.0231 | N.D. | di-galloyl hexoside V |
|  | 6.24 | 183.0298  (-5.336) | 185.0444  (-0.486) | C_8_H_8_O_5_ | **168.0052**, 151.0023, **124.0152** | 153.0182, **126.0313** | methyl gallate(Marcelino et al., 2023) |
|  | 6.59^#^ | 785.0844  (0.146) | N.D. | C_34_H_26_O_22_ | 633.0721, **300.9988, 275.0199, 249.0400,** | N.D. | di-galloyl-HHDP- hexoside |
|  | 6.80 | 635.0888  (-0.090) | N.D. | C_27_H_24_O_18_ | **465.0675**, **313.0563**, **169.0131**, **125.0227** | N.D. | tri-galloyl-hexoside II(Marcelino et al., 2023) |
|  | 7.46 | 935.0800  (-0.445) | N.D. | C_41_H_28_O_26_ | 783.0720, 633.0732, 463.0501, **300.9988, 275.0199, 249.0400, 169.0129** | N.D. | galloyl-di-HHDP-hexoside(Qiu et al., 2022) |
|  | 7.80 | 473.0367  (1.155) | 475.0503  (-0.835) | C_21_H_14_O_13_ | **321.0253**, **169.0131**, 125.0230 | N.D. | tri-gallic acid |
|  | 7.92 | 787.1003  (0.425) | 789.1135  (-1.316) | C_34_H_28_O_22_ | **635.0881**, **617.0797**, **465.0674**, **313.0563**, 295.0461, **169.0131**, 125.0230 | N.D. | tetra-galloyl hexoside(Cutovic et al., 2022) |
|  | 8.34 | 939.1109  (0.580) | N.D. | C_41_H_32_O_26_ | **769.0853**, **617.07867**,**447.05685**, 431.06223,295.04562, 277.03552, 169.0132 | N.D. | penta-galloyl hexoside(Cutovic et al., 2022) |
|  | 8.41 | 335.0408  (-0.165) | 337.0547  (-2.042) | C_15_H_12_O_9_ | **183.0289**, 168.0052, 124.0157 | **185.0443**, 153.0183 | methyl digallate I |
|  | 8.54 | 801.1144  (-2.129) | N.D. | C_35_H_30_O_22_ | **631.0939**, **479.0835**, 193.0136, **169.0132** | N.D. | methyl-tetra-galloyl hexoside I |
|  | 8.60 | 939.1109  (-0.004) | N.D. | C_41_H_32_O_26_ | **617.0779**,**465.0675**, **447.0570**, 431.0602, 313.0564, **295.0463**,277.0358,**169.0132** | N.D. | penta-galloyl hexoside II(Cutovic et al., 2022) |
|  | 8.63 | 625.0471  (-0.003) | N.D. | C_28_H_18_O_17_ | **321.0249**, **169.0132**, 125.0232 | N.D. | tetra-gallic acid(Cutovic et al., 2022) |
|  | 8.80 | 545.0576  (0.600) | N.D. | C_24_H_18_O_15_ | **393.0459**, **317.0401**, **169.0131** | N.D. | dihydroxybenzoic acetate-digallate |
|  | 8.80 | 801.1160  (0.468) | N.D. | C_35_H_30_O_22_ | **631.0933**, **479.0836**, **327.0718**,**183.0290**, **169.0132** | N.D. | methyl-tetra-galloyl hexoside II |
|  | 8.99 | 335.0414  (1.567) | 337.0546  (-2.398) | C_15_H_12_O_9_ | **183.0289**, 168.0054, 124.0150 | **185.0447**, 153.0182 | methyl digallate II |
| Flavonoids | | | | | | | |
|  | 5.44 | 801.2096  (0.118) | 803.2226  (-1.804) | C_34_H_42_O_22_ | **639.1565, 477.0991, 315.0498** | **317.0651,** 153.0184 | isorhamnetin-tri-hexoside I |
|  | 5.86 | 625.1410  (-0.052) | 627.1546  (-1.556) | C_27_H_30_O_17_ | **463.0870, 301.0351, 151.0024** | **303.0495** | quercetin-di-hexoside |
|  | 5.91 | 777.1512  (-1.031) | 779.1654  (-1.443) | C_34_H_34_O_21_ | **615.0986**, **463.0882**, **301.0345**, 169.0139 | N.D. | quercetin-(galloyl)-di-hexoside I |
|  | 6.42 | 609.1461  (0.020) | 611.1601  (-0.918) | C_27_H_30_O_16_ | **447.0927**, **285.0404**, **151.0021** | **449.1053**, **287.0549** | kaempferol-di-hexoside(Cutovic et al., 2022) |
|  | 6.50 | 817.2048  (0.513) | 819.2177  (-1.604) | C_34_H_42_O_23_ | **655.1520**, **493.0968**, **331.0438** | **495.1131**, **333.0598** | laricitrin-tri-hexoside |
|  | 6.54 | 639.1567  (0.106) | 641.1704  (-1.943) | C_28_H_32_O_17_ | **477.1031**, **315.0508** | **317.0652** | isorhamnetin-di-hexoside |
|  | 6.55 | 761.1571  (0.044) | 763.1700  (-2.070) | C_34_H_34_O_20_ | **599.1041**, **447.0925**, 313.0563, **285.0401**, **151.0029** | **449.1101**, **287.0548**, **153.0182** | luteolin-(galloyl) di-hexoside |
|  | 6.63 | 449.1089  (0.034) | 451.1232  (-0.705) | C_21_H_22_O_11_ | **287.0561**, 259.0612 | **289.0735** | dihydrokaempferol-hexoside I |
|  | 6.67 | 801.2103  (0.966) | 803.2230  (-1.281) | C_34_H_42_O_22_ | **639.1522**, **315.0496** | N.D. | Isorhamnetin-trihexoside II |
|  | 6.73 | N.D. | 463.1228  (-1.550) | C_22_H_22_O_11_ | N.D. | **301.0703**, 163.0600 | chrysoeriol-hexoside |
|  | 6.85 | 777.1533  (1.646) | 779.1625  (-2.093) | C_34_H_34_O_21_ | **615.0993**, **463.0672**, **301.0353**, 169.0131, 151.0025 | N.D. | quercetin-(galloyl)-dihexoside II |
|  | 6.82 | 631.0946  (0.836) | 633.1079  (-1.130) | C_28_H_24_O_17_ | **479.0836**, **317.0294** | **319.0445**, 153.0182 | myricetin-(galloyl)-hexoside |
|  | 6.87 | 695.1464  (-0.204) | 697.1597  (-1.901) | C_30_H_32_O_19_ | **447.0911**, **285.0406**, **489.1005** | **449.1075**, **287.0549**, 127.0390 | kaempferol-(malonyl)-dihexoside |
|  | 6.99 | 725.1572(0.226) | 727.1705  (-1.581) | C_31_H_34_O_20_ | **477.1008**, **315.0508**, **519.1142** | **317.0653** | isorhamnetin-(malonyl)-hexoside- hexoside |
|  | 7.06 | 447.0934  (0.146) | 449.1077  (-0.374) | C_21_H_21_O_11_ | **301.0711** | 153.0183 | quercetin-pentoside I |
|  | 7.10 | 593.1520  (1.326) | 595.1652  (-0.985) | C_27_H_30_O_15_ | **447.0894**, **285.0406** | **449.1062**, **287.0549**, **153.0183** | kaempferol-hexoside-deoxyhexoside |
|  | 7.11 | 449.1096  (1.392) | 451.1238  (0.581) | C_21_H_22_O_11_ | **287.0563**, 269.0457, 259.0613, **151.0023**, 125.0230 | N.D. | dihydrokaempferol-hexoside II |
|  | 7.14 | 655.1518  (0.310) | 657.1648  (-2.055) | C_28_H_32_O_18_ | **331.0431**, 315.0145, 209.0082 | **333.0598** | patuletin dihexoside |
|  | 7.15 | 609.1464  (0.414) | 611.1589  (-2.816) | C_27_H_30_O_16_ | **447.0915**, **301.0350**, **151.0027** | **303.0496** | quercetin-hexoside-deoxyhexoside |
|  | 7.23 | 761.1559  (-1.480) | 763.1729  (1.691) | C_34_H_34_O_20_ | **599.1047**, **447.0929**, **285.0405**, 151.0023 | **287.0548**, 153.0182, 127.0390 | kaempferol-(galloylhexoside)-hexoside |
|  | 7.38 | 801.2098  (0.355) | 803.2230  (-1.356) | C_34_H_42_O_22_ | **315.0498** | **317.0651** | isorhamnetin-trihexoside III |
|  | 7.36 | N.D. | 463.1229  (-1.226) | C_22_H_22_O_11_ | N.D. | **301.0704**, 163.0752, 153.0180 | Chrysoeriol-hexoside(Cutovic et al., 2022) |
|  | 7.48 | 615.0992  (0.134) | 617.1138  (0.096) | C_28_H_24_O_16_ | **463.0882**, **301.0346**, 169.0131, 151.0023 | **303.0493**, 153.0182 | Quercetin-(galloyl)-hexoside I |
|  | 7.57 | 639.1568  (0.199) | 641.1703  (-1.460) | C_28_H_32_O_17_ | **477.1064**, **315.0490** | **317.0652** | Isorhamnetin-dihexoside II |
|  | 7.58 | 593.1514  (0.298) | 595.1649  (-1.490) | C_27_H_30_O_15_ | **447.0936**, 431.0968, **285.0406** | **287.0548** | Kaempferol-hexoside-deoxyhexoside II |
|  | 7.65 | 669.1674  (0.273) | 671.1805  (1.658) | C_29_H_34_O_18_ | **345.0593**, 329.0299, **301.0343** | **347.0755** | Spinacetin-dihexoside |
|  | 7.70 | 623.1614  (-0.606) | 625.1749  (-2.209) | C_28_H_32_O_16_ | **477.1041**, **461.1081**, **315.0507**, 151.0029 | **317.0651** | Isorhamnetin-deoxyhexoside -hexoside |
|  | 7.75 | 609.1459  (-0.276) | 611.1599  (-1.327) | C_27_H_30_O_16_ | **447.0910**, **301.0319**, **151.0022** | **303.0496**, **153.0184** | Quercetin-deoxyhexoside-hexoside II |
|  | 7.87 | N.D. | 655.1869  (0.022) | C_29_H_34_O_17_ | N.D. | **347.0754** | [Syringetin-rutinoside](http://www.baidu.com/link?url=H2yeyhlKrhoPCp5qkOwXec17QAQdM-0GMHsy4AL2bJVpX2cL59dCYA05XDk81qkkJ8Fy6n7r4LL_f6EymbZRQ_9oi_RPVPSYH1GkbuU1sVW)(Mikulic-Petkovsek et al., 2012) |
|  | 7.91 | 725.1572  (0.143) | 727.1702  (-1.994) | C_31_H_34_O_20_ | **639.1599**, **618.1686**, **519.1150**, **315.0485**, 169.0131 | **317.0651** | Isorhamnetin-(malonyl)-dihexoside |
|  | 7.91 | 623.1617  (-0.029) | 625.1753  (-1.633) | C_28_H_32_O_16_ | **315.0475**, **169.5045** | **317.0651** | Isorhamnetin-deoxyhexoside -hexoside II |
|  | 7.94 | 549.0889  (0.504) | 551.1025  (-1.209) | C_24_H_22_O_15_ | **387.1567**, **301.0346**, 169.0133 | **303.0496** | (malonyl)-Quercetin-hexoside |
|  | 7.95 | 725.1571  (0.143) | 727.1702  (-1.994) | C_31_H_34_O_20_ | **619.9393**, **315.0485** | **317.0651** | Isorhamnetin-digallic acid-benzoic acid |
|  | 7.98 | 615.0992  (0.036) | 617.1132  (-0.893) | C_28_H_24_O_16_ | **463.0887**, **301.0350**, **151.0025** | **303.0495**, **153.0182** | Quercetin-(galloyl)-hexoside II |
|  | 8.01 | 653.1724  (0.149) | 655.1857  (-1.749) | C_29_H_34_O_17_ | **345.0580** | **347.0754** | [Syringetin-rutinoside](http://www.baidu.com/link?url=H2yeyhlKrhoPCp5qkOwXec17QAQdM-0GMHsy4AL2bJVpX2cL59dCYA05XDk81qkkJ8Fy6n7r4LL_f6EymbZRQ_9oi_RPVPSYH1GkbuU1sVW) (Mikulic-Petkovsek et al., 2012) |
|  | 8.04 | N.D. | 595.1282  (-1.900) | C_26_H_26_O_16_ | N.D. | **347.0756** | Syringetin-(malonyl)-hexoside |
|  | 8.07 | 755.1675  (-0.121) | 757.1809  (-1.683) | C_32_H_36_O_21_ | **593.1511, 447.0900, 285.0393** | **287.0578** | Kaempferol-rutinoside-hexoside |
|  | 8.17 | 623.1621  (0.565) | 625.1752  (-1.825) | C_28_H_32_O_16_ | **477.1016**, **315.0487**, 169.0133, 161.0219, 151.0025 | **463.1244**, **317.0652**, 153.0179 | Isorhamnetin-deoxyhexoside -hexoside III |
|  | 8.19* | 463.0885  (0.714) | 465.1022  (-1.188) | C_21_H_20_O_12_ | 343.0443, **301.0352**, 151.0024 | **303.0495**, 153.0180 | Hyperoside |
|  | 8.25* | 447.0924  (-2.023) | 449.1079  (0.094) | C_21_H_20_O_11_ | **285.0400**, 151.0026 | **287.0549**, 153.0182 | Luteoloside |
|  | 8.28* | 463.0889  (1.449) | 465.1025  (-0.586) | C_21_H_20_O_12_ | **301.0345**, 271.0253, 178.9976, 151.0026 | **303.0497** | Isoquercitrin |
|  | 8.47 | 767.1100  (-0.086) | 769.1238  (-1.156) | C_35_H_28_O_20_ | **615.0986**, **463.0886**, **301.0349**, 169.0130 | **303.0496**, 153.0182 | Quercetin-(digalloyl)-hexoside |
|  | 8.61 | 549.0889  (0.614) | 551.1027  (-0.882) | C_24_H_22_O_15_ | **387.0685**, **301.0336**, 169.0131 | **389.0862**, **303.0495** | (malonyl)-Quercetin-hexosideII |
|  | 8.61 | 505.0991  (0.705) | N.D. | C_23_H_22_O_13_ | **463.0869**, **301.0341**, 151.0024 | N.D. | Quercetin-acetylglucoside(Marcelino et al., 2023) |
|  | 8.69 | 599.1046  (0.512) | 601.1182  (-0.942) | C_28_H_24_O_15_ | **447.0935**, **285.0400**, 151.0031 | **287.0549**, **153.0182** | Kaempferol-(galloyl)-hexoside |
|  | 8.69**^#^** | 935.1528  (0.487) | N.D. | C_43_H_36_O_24_ | **599.1040**, **447.0919**, 335.0399, 313.0559, **285.0400**, 183.0289, 169.0129, 151.0028 | N.D. | kaempferol-(di-galloyl, methyl galloyl)-hexoside |
|  | 8.79 | 629.1146  (-0.298) | 631.1283  (-1.681) | C_29_H_26_O_16_ | **477.1042**, **315.0504**, **169.0132**, 151.0026 | **317.0651**, **171.0286**, 153.0181 | Isorhamnetin-(galloyl)-hexoside |
|  | 8.86* | 447.0935  (0.571) | 449.1074  (-1.064) | C_21_H_20_O_11_ | **285.0389**, 255.0294, 151.0026 | **287.0549** | Astragalin |
|  | 8.89* | 303.0512  (0.706) | 305.0653  (-1.079) | C_15_H_12_O_7_ | **151.0027** | **153.0182** | Taxifolin |
|  | 8.92 | 477.1039  (0.190) | 479.1181  (-0.694) | C_22_H_22_O_12_ | **315.0484**, 151.0025 | **317.0652** | Isorhamnetin hexoside |
|  | 9.00 | N.D. | 433.1125  (0.270) | C_21_H_20_O_10_ | N.D. | **271.0600**, 153.0181, 137.0592, 72.6734 | Apigenin hexoside |
|  | 9.01 | 447.0933  (-0.055) | 449.1072  (-1.000) | C_21_H_20_O_11_ | **285.0403**, 151.0024 | **287.0562**, 153.0181 | Kaempferol-hexoside |
|  | 9.02 | 751.1287  (-1.374) | 753.1286  (1.454) | C_35_H_28_O_19_ | **599.1041**, **447.0936**, **285.0399** | **449.0720**, **287.0550**, 153.0183 | Kaempferol-(di-galloyl)-hexoside |
|  | 9.10 | 477.1039  (0.127) | 479.1180  (-0.944) | C_22_H_22_O_12_ | **315.0500**, 151.0024 | **317.0652** | Isorhamnetin hexoside II |
|  | 9.25 | 489.1043  (0.942) | N.D. | C_23_H_22_O_12_ | **327.0515**, **285.0401**, 151.0026 | N.D. | Kaempferol-acetyl-hexoside |
|  | 9.27 | 533.0938  (0.134) | 535.1075  (-1.293) | C_24_H_22_O_14_ | **285.0402** | **373.0934**, **287.0547** | Kaempferol-malonyl-hexoside I |
|  | 9.35 | 599.1047  (0.729) | 601.1186  (-0.343) | C_28_H_24_O_15_ | **447.0944**, **285.0404**, 169.0131, 151.0026 | **287.0549**, **171.0286**, 153.0182 | Kaempferol-galloyl-hexoside II |
|  | 9.37 | 563.1038  (-0.751) | 565.1182  (-1.002) | C_25_H_24_O_15_ | **315.0510** | **359.0747**, **317.0652** | Isorhamnetin-malonyl-hexoside |
|  | 9.37 | 519.1143  (-0.296) | N.D. | C_24_H_24_O_13_ | **357.0607**, **315.0499**, 271.0254, 151.0025 | N.D. | Isorhamnetin-(acetyl)-hexoside |
|  | 9.45 | 533.0936  (-0.203) | 535.1077  (-0.938) | C_24_H_22_O_14_ | **285.0405** | **373.0934**, **287.0547** | Kaempferol-malonyl-hexoside II |
|  | 9.55 | 447.0930  (-0.659) | 449.1077  (-0.374) | C_21_H_20_O_11_ | **301.0346**, 151.0021 | **303.0493**, 153.0182 | Quercetin-pentoside II |
|  | 9.82 | 431.0984  (0.046) | 433.1129  (-0.169) | C_20_H_20_O_10_ | **285.0380**, 151.0023, 125.0225 | **287.0548**, 153.0192 | Luteolin-deoxyhexoside I |
|  | 9.96 | 287.0557  (-1.328) | 289.0707  (0.157) | C_15_H_12_O_6_ | 259.0613, 243.0659, **151.0022**, **125.0230** | N.D. | Dihydrokaempferol |
|  | 10.50 | 431.0985  (0.186) | 433.1125  (-1.070) | C_21_H_20_O_10_ | **285.0403**, 151.0025, 145.0282 | **287.0550**, 153.0179 | Luteolin-deoxyhexoside II |
|  | 10.59 | 623.1405  (-0.206) | 625.1540  (-1.907) | C_31_H_28_O_14_ | **477.1041**, **315.0504**, 169.0131, 151.0023 | **463.5930**, **317.0651**, 153.0184 | Isorhamnetin-(*p*-coumaroyl)-hexoside |
|  | 10.98* | 301.0349  (-1.548) | 303.0496  (-1.020) | C_15_H_10_O_7_ | 273.0407, 257.0447, 229.0502, 178.9973, **151.0023**, 121.0280 | **153.0185** | Quercetin |
|  | 11.26 | N.D. | 463.1593  (-1.303) | C_23_H_26_O_10_ | N.D. | **301.0706**, 179.0705, 121.0284, 105.0339 | Hydroxy-dimethoxy -flavanone-O-hexoside |
|  | 11.97* | 269.0451  (-1.660) | 271.0603  (-0.147) | C_15_H_10_O_5_ | 225.1488, **151.4542** | N.D. | Apigenin |
|  | 12.03* | 271.0611  (-0.357) | 273.0759  (0.659) | C_15_H_12_O_5_ | 177.0181, **151.0024** | **153.0182** | Naringenin |
|  | 12.21* | 285.0407(0.873) | 287.0553  (1.099) | C_15_H_10_O_6_ | 257.0453, **151.0024**, 107.7064 | **153.0177** | Kaempferol |
| **Anthocyanins** | | | | | | | |
|  | 3.14* | 609.1462  (1.019) | 611.1601  (-1.033) | C_27_H_31_O_16_ | N.D. | **449.1094**, **287.0550** | Cyanidin-3,5-O-diglucoside |
|  | 4.33 | 593.1.489  (-3.009) | 595.1655  (-1.907) | C_27_H_31_O_15_ | N.D. | **433.1120**, **271.0602** | Pelargonidin-dihexoside |
|  | 4.97* | 623.1611  (-0.224) | 625.1751  (-2.799) | C_28_H_33_O_16_ | N.D. | **463.1227**, **301.0705** | Peonidin-3,5-O-diglucoside |
|  | 4.93* | 463.0875  (-0.282) | 465.1024  (-1.894) | C_21_H_21_O_12_ | N.D. | **303.0486**, **153.0182** | [Delphinidol-3-O-glucoside](https://www.chemicalbook.com/Search.aspx?keyword=Delphinidol%203-glucoside) |
|  | 5.86* | 447.0925  (-0.461) | 449.1073  (-2.419) | C_21_H_21_O_11_ | **285.0399** | **287.0548**, 153.0181 | Cyanidin-3-O-glucoside |
|  | 5.93* | N.D. | 479.1196  (1.355) | C_22_H_23_O_12_ | N.D. | **317.0652**, 121.0801, 97.0290 | Petunidin-3-O-glucoside |
|  | 6.09* | N.D. | 595.1659  (-0.680) | C_27_H_31_O_15_ | N.D. | **287.0549** | Cyanidin-3-O-rutinoside |
|  | 6.56* | N.D. | 433.1140  (1.312) | C_21_H_21_O_10_ | N.D. | N.D. | Pelargonidin-3-O-glucoside |
|  | 6.59* | 461.1074  (-2.095) | 463.1229  (-2.475) | C_22_H_23_O_11_ | **299.0563**, **161.0445** | **301.0703**, 286.0471 | Peonidin-3-O-glucoside |
|  | 6.63* | N.D. | 493.1338  (-1.888) | C_23_H_25_O_12_ | N.D. | N.D. | Malvidin-3-O-glucoside |
|  | 6.89* | N.D. | 535.1088(0.093) | C_24_H_23_O_14_ | N.D. | **287.0548** | [Cyanidin 3-(6”-malonylglucoside)](http://www.baidu.com/link?url=FFJi5wn3T21pHsfh6HkSxPAiUkgjd9r20UiNRvt8sW1fc5cQaDzzhnVcdCbA2ahL-RneOMjk7HYFKL9M918RCYeE4p9zGSe7WURe4o-4d4C) |
|  | 6.92* | N.D. | 303.0496  (-1.119) | C_15_H_11_O_7_ | N.D. | **153.0188** | Delphinidin |
|  | 7.63* | N.D. | 287.0548  (-2.727) | C_15_H_11_O_6_ | N.D. | 229.0970, 121.0288 | Cyanidin |
|  | 7.73* | N.D. | 317.0649  (-3.840) | C_16_H_13_O_7_ | N.D. | 302.0418, 72.6339 | Petunidin |
|  | 8.26* | N.D. | 271.0613  (2.220) | C_15_H_11_O_5_ | N.D. | **153.0183** | Pelargonidin |
|  | 8.44* | N.D. | 331.0806  (-3.557) | C_17_H_15_O_7_ | N.D. | 287.1273, 179.0341, 137.0596, 114.0917, 73.0317 | Malvin |
| **Monoterpene glycosides** | | | | | | | |
|  | 4.38 | 345.1185  (-1.841) | N.D. | C_15_H_22_O_9_ | **183.0288**, 165.0183, **161.0183**, 147.2568, 119.0332 | N.D. | Aucubin |
|  | 5.20 | 495.1514  (1.152) | 497.1648  (-1.051) | C_23_H_28_O_12_ | **465.1385**, 281.0667, 195.0658, 169.0450, **137.0231**, 93.0332 | 283.0819, 197.0806, 139.0389 | Oxypaeoniflora I |
|  | 5.70 | 495.1512  (0.829) | N.D. | C_23_H_28_O_12_ | **465.1401**, 345.1194, **333.0982**, 281.0673, 195.0651, **165.0546**, **137.0231**, 93.0330 | N.D. | Oxypaeoniflora II |
|  | 6.16 | 525.1617  (0.602) | 527.1745  (-2.689) | C_24_H_30_O_13_ | **495.1502**, **363.1064**, **329.0860**, **167.0338**, **165.0543** | N.D. | Mudanpioside E |
|  | 6.72 | 641.2091  (0.627) | 643.2220  (-1.992) | C_29_H_38_O_16_ | **489.1655**, **121.0281** | N.D. | Isomaltopaeoniflorin |
|  | 6.86 | 495.1511  (0.647) | N.D. | C_23_H_28_O_12_ | 345.1186, **333.0962**, 281.0670, 195.0654, **165.0547**, **137.0231** | N.D. | Oxypaeoniflora III |
|  | 7.17 | 641.2089  (0.237) | 643.2218  (0.480) | C_29_H_38_O_16_ | **489.1611**, **177.0546**, **121.0281** | 197.0806, **179.0703** | Albiflorin hexoside |
|  | 7.17 | 479.1555  (-0.699), 525.1616  [M+HCOOH]^-^ | 481.1701  (-0.619) | C_23_H_28_O_11_ | **449.1427**, **357.1210**, **327.1077**,  **195.0657**, 165.0546,121.0281 | 197.0806, **179.0703**, 151.0754, 105.0339 | Albiflorin |
|  | 7.47 | 495.1509  (0.284) | 497.1652  (-0.388) | C_23_H_28_O_12_ | 345.1189, 281.0668, 195.0650, **137.0231** | 197.0811, **179.0702**, 151.0754, 121.0286 | oxypaeoniflora IV |
|  | 7.53* | 479.1560  (0.324)  , 525.1620  [M+HCOOH] | 481.1704  (-0.121) | C_23_H_28_O_11_ | **327.1077**, **165.0546**, **121.0281** | **179.0703**, 167.0347, 151.0754 | paeoniflorin |
|  | 8.47* | 631.1669  (0.137) | 633.1802  (-1.827) | C_30_H_32_O_15_ | **613.1542**, **491.1201**,465.1400, 399.0924, 313.0562, **271.0239**, **211.0239**, 169.0131 | 297.0613, 237.0396, 167.0338, 153.0181, 105.0339 | galloylpaeoniflorin I |
|  | 8.79 | 615.1712  (-1.185) | N.D. | C_30_H_32_O_14_ | **585.1592**, **477.1409**, 447.1313, 281.0666, 239.0554, 137.0230 | N.D. | mudanpioside H |
|  | 8.81 | 631.1677  (1.389) | N.D. | C_30_H_32_O_15_ | 465.0.934, 399.0957, 313.0565, **271.0462**, **211.0242**, 169.0132 | N.D. | galloylpaeoniflorin II |
|  | 9.92 | 599.1771  (0.177) | 601.1907  (-1.493) | C_30_H_32_O_13_ | **431.1354**, **281.0667**, **165.0543**, **137.0230** | N.D. | benzoyloxypaeoniflflorin |
|  | 10.43 | 599.1774  (0.694) | 601.1923  (-0.478) | C_30_H_32_O_13_ | **477.1402**, **447.0916**, **165.0545** | 197.0808, 139.0384 | mudanpioside C |
|  | 11.27 | 629.1876  (-0.030) | N.D. | C_31_H_34_O_14_ | **165.0544**, **121.0281** | N.D. | mudanpioside J |
|  | 11.47 | 629.1874  (-0.316) | N.D. | C_31_H_34_O_14_ | 541.5765, **165.0549**, **121.0280** | N.D. | mudanpioside B |
|  | 4.87^#^ | 527.1409  (0.458) | 529.1550  (-0.287) | C_23_H_28_O_14_ | **375.1293**, 345.1187, **313.0562**, **271.0462**,**169.0131**, **151.0020**, | N.D. | galloyl desbenzoyl albiflorin |
|  | 5.86^#^ | 527.1405  (-0.244) | 529.1555  (0.639) | C_23_H_28_O_14_ | **375.1292**, 345.1187, **313.0561**, **169.0130**, **151.0023**, **271.0462** | N.D. | galloyl desbenzoyl paeoniflorin |
| **Other phenolics** | | | | | | | |
|  | 2.84 | 315.0725  (1.126) | N.D. | C_13_H_16_O_9_ | **153.0181**, 109.0281 | N.D. | protocatechuic acid hexoside I |
|  | 3.17 | 299.0772  (-0.303) | N.D. | C_13_H_16_O_8_ | **179.0338, 137.0231** | N.D. | *p*-hydroxybenzoic acid hexoside |
|  | 3.22 | 315.0722  (0.142) | N.D. | C_13_H_16_O_9_ | **153.0181**,109.0280 | N.D. | protocatechuic acid hexoside II |
|  | 3.52 | 355.0667  (-0.929) | 357.0827  (3.100) | C_15_H_16_O_10_ | **209.0295**, **191.0186**, **163.0382**, 147.0285, 85.0279 | N.D. | *p*-coumaryl glucarate I |
|  | 4.23 | 313.0563  (-0.528) | 315.0703  (-2.280) | C_13_H_14_O_9_ | **191.0187**, 147.0284, 129.0179, 85.0279 | 153.0182 | Benzoic acid glucarate I |
|  | 4.64 | 355.0667  (-1.013) | 357.0821  (1.392) | C_15_H_16_O_10_ | **209.0295**,**191.0188**,**163.0390**,147.0288,85.0280 | N.D. | *p*-coumaryl glucarate II |
|  | 5.29 | 355.0668  (-0.844) | 357.0813  (-0.812) | C_15_H_16_O_10_ | **209.0294**, **191.0187**, **163.0394**, 147.0286, 85.0279 | N.D. | *p*-coumaryl glucarate III |
|  | 5.38 | 313.0563  (-0.528) | 315.0706  (-1.392) | C_13_H_14_O_9_ | **191.0185**,147.0286,129.0179,85.0279 | 153.0182 | benzoic acid glucarate II |
|  | 5.66 | 313.0565  (-0.144) | 315.0707  (-1.106) | C_13_H_14_O_9_ | **191.0187**, 147.0287, 129.0179, 85.0280 | 153.0182 | benzoic acid glucarate III |
|  | 5.69 | 137.0242  (-1.951) | 139.0390  (-0.652) | C_7_H_6_O_3_ | **93.0331**, 73.3307 | 121.0286**, 95.0497** | hydroxybenzoic acid |
|  | 6.66 | 313.0564  (-0.240) | 315.0703  (-2.280) | C_13_H_14_O_9_ | **191.0187**, 147.0287, 129.0181, 85.0280 | 153.0182 | benzoic acid glucarate IV |
|  | 6.78 | 415.1606  (-1.012)  461.16711  [M+HCOOH]^-^ | 417.1747  (-2.094) | C_19_H_28_O_10_ | **269.1032**,**161.0443**,143.0342, 113.0232,101.0230 | **145.0494** | hydlrageifolin I |
|  | 8.08 | 391.1033  (-0.321) | 393.1164  (-4.041) | C_19_H_20_O_9_ | **281.0668**, **137.0231**, **109.0281**,93.0331 | N.D. | hydroxybenzoyl arbutin |
|  | 9.60 | 435.0931  (-0.332) | N.D. | C_20_H_20_O_11_ | **273.0770** | N.D. | glucosy-dihydroxy-methoxyxanthone |

* Targets were quantified by using reference compounds.

**Reference**

Cutovic, N., Markovic, T., Kostic, M., Gasic, U., Prijic, Z., Ren, X., et al. (2022). Chemical Profile and Skin-Beneficial Activities of the Petal Extracts of Paeonia tenuifolia L. from Serbia. *Pharmaceuticals (Basel)* 15(12). doi: 10.3390/ph15121537.

Marcelino, S., Mandim, F., Taofiq, O., Pires, T., Finimundy, T.C., Prieto, M.A., et al. (2023). Valorization of Punica granatum L. Leaves Extracts as a Source of Bioactive Molecules. *Pharmaceuticals (Basel)* 16(3). doi: 10.3390/ph16030342.

Mikulic-Petkovsek, M., Slatnar, A., Stampar, F., and Veberic, R. (2012). HPLC-MSn identification and quantification of flavonol glycosides in 28 wild and cultivated berry species. *Food Chem* 135(4)**,** 2138-2146. doi: 10.1016/j.foodchem.2012.06.115.

Milczarek, A., Sojka, M., and Klewicki, R. (2021). Transfer of ellagitannins to unclarified juices and purees in the processing of selected fruits of the Rosaceae family. *Food Chem.* 344**,** 128684. doi: 10.1016/j.foodchem.2020.128684.

Qiu, J., Chen, X., Liang, P., Zhang, L., Xu, Y., Gong, M., et al. (2022). Integrating approach to discover novel bergenin derivatives and phenolics with antioxidant and anti-inflammatory activities from bio-active fraction of Syzygium brachythyrsum. *Arabian Journal of Chemistry* 15(1). doi: 10.1016/j.arabjc.2021.103507.
